# Quantifying the origin of metallic glass formation

**DOI:** 10.1038/ncomms10313

**Published:** 2016-01-20

**Authors:** W. L. Johnson, J. H. Na, M. D. Demetriou

**Affiliations:** 1 Keck Laboratory of Engineering, Department of Materials Science, 138-78 California Institute of Technology, Pasadena, California 91125, USA; 2 Glassimetal Technology Inc., 2670 Walnut St. Ave., Pasadena, California 91107, USA

## Abstract

The waiting time to form a crystal in a unit volume of homogeneous undercooled liquid exhibits a pronounced minimum *τ*_X_* at a ‘nose temperature' T^*^ located between the glass transition temperature *T*_g_, and the crystal melting temperature, *T*_L_. Turnbull argued that *τ*_X_* should increase rapidly with the dimensionless ratio *t*_rg_=*T*_g_/*T*_L_. Angell introduced a dimensionless ‘fragility parameter', *m*, to characterize the fall of atomic mobility with temperature above *T*_g_. Both *t*_rg_ and *m* are widely thought to play a significant role in determining *τ*_X_*. Here we survey and assess reported data for *T*_L_, *T*_g_, *t*_rg_, *m* and *τ*_X_* for a broad range of metallic glasses with widely varying *τ*_X_*. By analysing this database, we derive a simple empirical expression for *τ*_X_*(*t*_rg_, *m)* that depends exponentially on *t*_rg_ and *m*, and two fitting parameters. A statistical analysis shows that knowledge of *t*_rg_ and *m* alone is therefore sufficient to predict *τ*_X_* within estimated experimental errors. Surprisingly, the liquid/crystal interfacial free energy does not appear in this expression for *τ*_X_*.

## Abstract (web summary)

The crystal formation waiting time of a homogeneous supercooled liquid exhibits a pronounced minimum depending on temperature, and this minimum is a measure of glass forming ability. Here, the authors propose an expression for this minimum for all metallic glasses that depends only on two fitting parameters.

## Graphical abstract

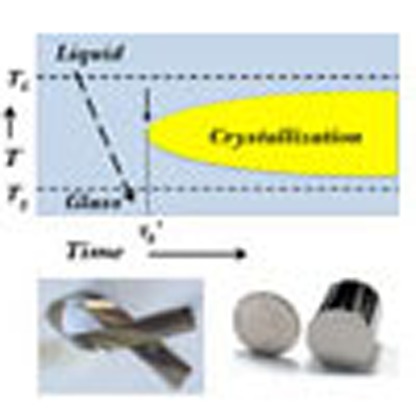

It is widely believed that any liquid will form a glass if cooled sufficiently rapidly. Even elemental liquid metals can be vitrified^1^,^2^ if quenched to low temperature at ultrahigh cooling rates of 10^12^–10^14^ K s^−1^. At the opposite extreme are certain molten eutectic alloys that form bulk metallic glass at cooling rates of ∼1 K s^−1^ or less^3^,^4^. The glass forming ability (GFA) of a liquid is defined by the temperature-dependent waiting time, *τ*_X_(*T*), for a detectable fraction of crystal(s) to nucleate and grow in a unit volume of liquid undercooled to a temperature *T*<*T*_L_, where *T*_L_ is the melting temperature or more specifically the liquidus temperature of an alloy^5^,^6^,^7^,^8^. Far below *T*_L_, liquids undergo configurational freezing at the glass transition temperature *T*_g_ and crystal nucleation is kinetically arrested. Between *T*_g_ and *T*_L_, the *τ*_X_(*T*)-curve, or so-called time–temperature–transformation diagram (TTT diagram), exhibits a sharp minimum *τ*_X_* at an experimentally measureable nose temperature T* (refs 5, 6, 7). To form glass, the liquid must roughly be cooled from *T*_L_ to below T* in a time less than *τ*_X_*. This defines a critical cooling rate *R*_C_∼[*T*_L_−T*]/*τ*_X_* that must be exceeded to avoid crystallization. Transient cooling, or quenching, is often governed by time-dependent heat conduction. Heat is extracted from the external sample boundary; and the cooling history is a function of location within the sample, being highest near the surface and lowest near the sample center of symmetry (for example, along the centerline of a rod or mid-plane of a plate). For a uniform shape with characteristic sample dimension *d* (for instance, a rod diameter or plate thickness), a Fourier time scale or thermal relaxation time can be defined as *τ*_Q_∼*d*^2^/*D*_t_, where *D*_t_ is the liquid thermal diffusivity and is roughly constant among the various metallic glass alloy compositions (typically 2–4 mm^2^ s^−1^). This time scale characterizes cooling at the center of symmetry. The proportionality constant depends on the geometry. Requiring *τ*_Q_<*τ*_X_* implies a maximum sample dimension *d*_max_ for forming a glass referred to as the critical casting thickness of the alloy. The parameters *τ*_X_*, *R*_C_ and *d*_max_ are interchangeably used in the literature as alternative measures of GFA.

Turnbull and others^5^,^6^,^7^,^8^ showed generally that the nucleation rate can be expressed as the product of a pre-factor and two thermally activated rate factors:


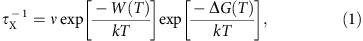


where the pre-factor *v* is taken to be a typical atomic vibration frequency. The first exponential factor is the thermally activated atomic rearrangement rate in the liquid. It describes atomic mobility, fluidity or inverse viscosity *η*^−1^∼exp[−*W*(*T*)/*kT*]. The atomic rearrangement barrier *W*(*T*) falls with increasing *T* above *T*_g_. To characterize the rate of fall of *W(T)*, Angell introduced the concept of liquid fragility that he quantified using a fragility parameter, *m* (refs 9, 10). Viscosity is modelled by various empirical laws^11^,^12^,^13^,^14^,^15^. The most common is the Vogel-Fulcher-Tamann law, though recently a more reliable law based on the cooperative shear flow model has been introduced that is directly relatable to the definition of *m* (refs 13, 14) (Supplementary Information).

The second factor exp[−Δ*G*/*kT*] in equation (1) is the probability of a fluctuation leading to the formation of a critical crystalline nucleus. In Classical Nucleation Theory (CNT), Δ*G*(*T*), arises from the excess free energy per unit area, *γ*_XL_, required to form the liquid/crystal interface^5^,^6^,^7^,^8^. As the temperature *T* of an undercooled liquid increases approaching *T*_L_, Δ*G*(*T*) increases rapidly with *T* and diverges to infinity as (*T*_L_−*T*)^−2^ so that the nucleation rate becomes immeasurably small^5^,^6^ near *T*_L_. The temperature dependence of the sum *W*(*T*)+Δ*G*(*T*) is dominated by the rapid drop of *W*(*T*) for *T*>*T*_g_, and by the divergence of Δ*G* as *T*→*T*_L._ This sum therefore exhibits a sharp minimum at some intermediate temperature T*, where *τ*_X_(T*) exhibits a pronounced minimum. CNT, therefore, correctly predicts the expected ‘C'-shaped TTT diagram.

Turnbull^5^ suggested that if *t*_rg_>2/3, then *τ*_X_* should exceed typical experimental time scales (for example, 1–10^3^ s) thereby resulting in easy glass formation. Metallic glasses were first synthesized by rapid quenching (cooling rates ∼10^6^ K s^−1^) of low-melting eutectic Au–Si and Pd–Si alloys by Duwez and colleagues^16^,^17^. For a eutectic alloy^5^,^6^,^18^, the composition-dependent *T*_L_ curve forms a cusp-like minimum at the eutectic composition and *t*_rg_ exhibits a corresponding cusp-like maximum. For a particularly low melting ternary near-eutectic alloy, Pd_40_Ni_40_P_20_, with *t*_rg_≈0.6, Turnbull's group used fluxing methods to purify the liquid and cast bulk glassy ingots with *d*_max_≈1 cm (refs 19, 20).

It has been argued that strong liquids (with low *m*) should exhibit greater GFA than fragile liquids (with high *m* values)^21^,^22^,^23^,^24^. For example, Mukherjee *et al.* demonstrated that within a limited group of Zr-based alloys, the variation in viscosity at the measured T* of the TTT diagram scaled with the measured variation in *τ*_X_* (ref. 22). Senkov attempted to quantify the role of *m* (ref. 23) in GFA of metallic glasses by assuming *τ*_X_* to be proportional to liquid viscosity at T*. Estimating T* to be roughly the average of *T*_g_ and *T*_L_, he expressed *η*(T*), in terms of *t*_rg_ and *m* and further assumed that crystal nucleation times for metallic glasses are simply proportional to *η*(T*) or equivalently to a parameter *F*_*1*_=2[(*m*/16)(*t*_rg_^−1^−1)+2]^−1^, introduced by Senkov to estimate *η*(T*). Na *et al.*^24^ recently reported detailed maps of *d*_max_ (or equivalently *τ*_X_*) as a function of composition **c** of a five-component Ni–Cr–Nb–P–B alloy where **c** represented a vector composition variation near a eutectic composition. They demonstrated that these maps could be quantitatively understood in terms of measured composition variations *t*_rg_(**c**) and *m*(**c**). They suggested that their analysis might be generalized to other alloys^24^.

In the present work, we conduct a broad survey and critical assessment of the published literature on metallic glasses. We have compiled a database that includes values of *d*_max,_ *τ*_X_*, *T*_g_, *T*_L,_ *t*_rg,_ and fragility parameter *m* for broad range of metallic glasses with widely varying GFA. Based on an analysis of this database, we introduce a simple empirical expression that assumes log(*τ*_X_*) to be a bilinear function of *t*_rg_ and *m*. Fitting this expression to our database captures the systematics of *τ*_X_* in real metallic glasses within estimated experimental uncertainties in the relevant parameters alone. Our result shows that the maximum crystallization rate in undercooled metallic liquids can be rationalized using a remarkably simple picture. We discuss the implications of this finding in the context of traditional nucleation theory.

## Results

### Compiled database for metallic glasses

Supplementary Table I contains a summary of experimental data for the critical casting diameter, *d*_max_, along with data for *τ*_X_*, *T*_g_, *T*_L_, *t*_rg_ and *m*, for ∼40 diverse but well-characterized metallic glass-forming alloys that exhibit widely varying GFA. The tabulated data are obtained from a comprehensive and critical assessment of published literature on metallic glasses. References and details of methods used to evaluate published data are provided in the Supplementary Information together with a discussion of experimental errors in the data. The alloys included in this database were selected based on the availability of reliable, reproducible and consistently determined values for GFA and the other relevant parameters. The criteria used to assess the reported experimental data are described. The discussion includes a comparison of alternate experimental measures of GFA, for example, *τ*_X_* versus *d*_max_. A standardized approach was used to determine consistent values for *t*_rg_ and *m* from calorimetric and rheological data. For instance, the tabulated values of *T*_g_ are based on the rheological definition of the glass transition, *η*(*T*_g_)=10^12^ Pa-s. Determining reliable *m* values requires equilibrium liquid viscosity data in the vicinity of the rheological *T*_g_ (typically between *η*∼10^7^ and 10^13^ Pa s^−1^). Data at high temperatures (near or above *T*_L_ where *η*∼0.001–1 Pa s^−1^) were used where available and combined with the low temperature data to obtain a best value of *m* for each alloy. Viscosity analysis based solely on high-temperature data is found to yield consistently large errors in and overestimates of *m* (compared with low temperature data) and is, therefore, not used for quantitative analysis (as discussed in the Supplementary Information).

### Correlation of GFA with Turnbull's *t*_rg_ or Angell's *m* alone

**Figure 1:**
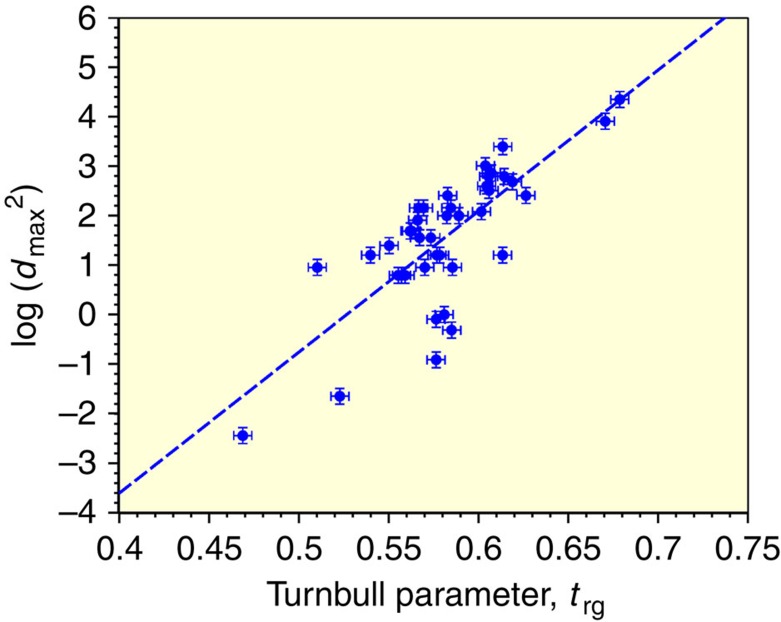
Plot of log(*d*_max_^2^) versus the dimensionless parameter *t*_rg_. Dashed line is the result of a linear regression least squares fit with an average slope of *Λ*_t_=28.5. The coefficient of determination for the linear fit is *R*^2^ is 0.598. Error bars are estimated expeimental errors as described in the text.

The correlation between Turnbull's *t*_rg_ and GFA (defined by either *τ*_X_* or *d*_max_^2^), is illustrated by plotting either log(*τ*_X_*) or log(*d*_max_^2^) versus *t*_rg_ for the database (Supplementary Table I in Supplementary Information). This is shown in Fig. 1. The plot shows an expected trend that can be described by a linear regression with an average slope of Λ_t_=*d*[log(*d*_*max*_^2^)]/*dt*_rg_=28.5, as illustrated. The linear form implies an exponential dependence of *τ*_X_* or equivalently *d*_max_^2^ on *t*_rg_ of the form *τ*_X_*∼exp(Λ_t_*t*_rg_), as might be anticipated from equation (1). While a trend is clear, the scatter in the plot is large. The relative scatter is quantified by the coefficient of determination *R*^2^=0.598 for the linear fit. The assumed linear correlation accounts for ∼60% of the total variance in the log(*d*_max_^2^) values, or equivalently the log(*τ*_X_*) values. The mean square misfit (per data point) is ∼0.96 yielding a standard error or s.e. of ±0.98 in log(*d*_max_^2^). Apparently, *t*_rg_ alone predicts *τ*_X_* within roughly plus/minus one order of magnitude. This uncertainty should be contrasted with the overall variation of *τ*_X_* over ∼7–8 orders of magnitude over the database. One concludes that *t*_rg_, while useful, is an inadequate quantitative predictor of GFA.

**Figure 2 f2:**
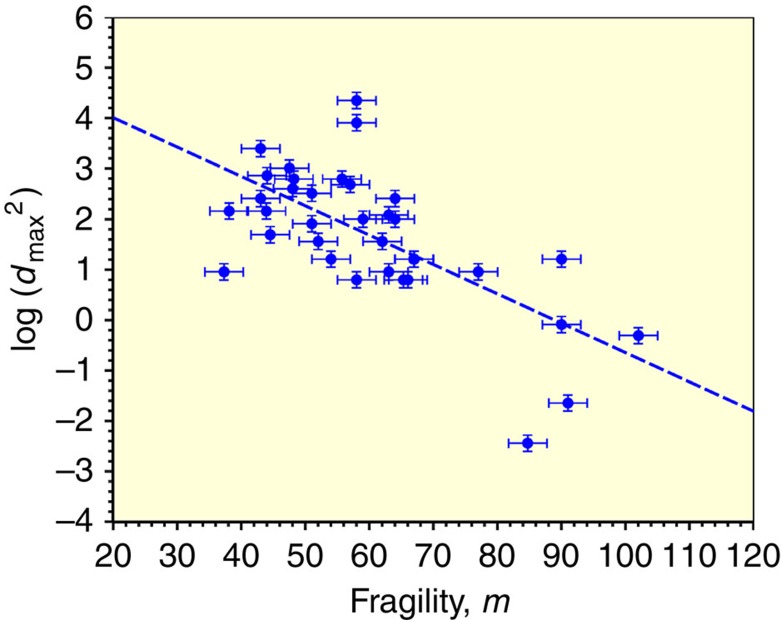
Plot of log(*d*_max_^2^) as a function of Angell's fragility parameter *m*. Dashed line is the result of a linear regression least squares fit with an average slope of *Λ*_m_=−0.0572. The coefficient of determination *R*^2^ is 0.458. Error bars are estimated experimental errors in fragility and casting diameter as described in the text.

Figure 2 plots the variation of log(*d*_max_^2^), or equivalently log(*τ*_X_*), versus Angell's parameter *m* for the systems in Supplementary Table I. As was the case for Fig. 1, a trend is clearly visible. Linear regression yields an average slope *Λ*_m_=−0.0572 with a coefficient of determination of *R*^2^=0.458. The correlation of GFA with *m* alone accounts for ∼46% of the variance in the experimental values of log(*d*_max_^2^). The standard error of the misfit in log(*d*_max_^2^) is ∼1.26, similar to but somewhat greater than that obtained from the correlation with *t*_rg_ in Fig. 1. Angell's parameter alone actually correlates with observed GFA nearly as well as *t*_rg_ alone. Summarizing, either *m* or *t*_rg_ alone can be used to predict *τ*_X_* within roughly plus/minus one order of magnitude. The two parameters, *t*_rg_ and *m*, are apparently of roughly equal utility in predicting GFA. For comparison with Figs 1 and 2, we have included a plot of Senkov's *F*_*1*_ versus log(*d*_max_^2^) for our database. This is shown in the Supplementary Fig. 2 for reference. Senkov's parameter depends on both *t*_rg_ and *m* and provides an improved correlation with experimental GFA compared with either Figs 1 or 2, [Fig f2]. A linear regression gives *R*^2^=0.879, a significantly improved description of GFA than provided by either *t*_rg_ or *m* alone. As will shortly be seen, one can do much better without making arbitrary assumptions regarding the location of T* with respect to *T*_g_ and *T*_L_.

### A bilinear expression for log(*τ*_X_*) in terms of both *t*_rg_ and *m*

In the work of Na *et al.*^24^, it was demonstrated that the GFA-composition variation around a deep eutectic composition takes the form of exponential hyper-cusps in the four-dimensional composition space of the five-component Ni–Cr–Nb–P–B alloy. Using the measured composition dependences of *t*_rg_ and *m*, they introduced a bilinear expression for 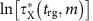 to interpret the experimental GFA-composition variation. The fitting parameters *Λ*_t_=d[ln(*d*_max_^2^)]/d*t*_rg_ and *Λ*_m_=d[ln(*d*_max_^2^)]/d*m* were taken as characteristic of the Ni–Cr–Nb–P–B alloy system. Their analysis yielded values *Λ*_t_ =89±20% and *Λ*_m_≈−0.2±40%. (errors are estimated). The authors noted that their expression for 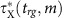 might be more generally applicable. The similarity of their *Λ*_t_ with that obtained from the linear fit in Fig. 1, *Λ*_t_=28.5 × ln(10)∼66 of is suggestive (Note, the factor of ln(10) arises from the present use of the log(*d*_max_^2^) versus ln(*d*_max_^2^) in ref. 24).

Following ref. 24, we shall assume that log(*τ*_X_*), or equivalently log(*d*_max_^2^), is a bilinear function of the two independent variables *t*_rg_ and *m*. Whereas Na *et al.* applied their equation to near-eutectic alloys of a single system (Ni–Cr–Nb–P–B), we apply it to all glass forming alloys in our database. We assume, quite generally, that log(*d*_max_^2^) is some continuous and differentiable function of the independent variables *t*_rg_ and *m*. In principle, this function might also depend on other independent material parameters besides *t*_rg_ and *m*. For instance, the interfacial free energy of a liquid/crystal interface, *γ*_XL_, is a natural third parameter arising in CNT. Here we test the assumption that log(*d*_max_^2^) is a universal function of *t*_rg_ and *m* alone. Consider the expansion of log(*d*_max_^2^) in a Taylor series around some reference values (*t*_rg,0_, *m*_0_). To lowest order, one has:


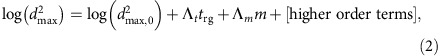


where the reference values have, without loss of generality, been set equal to zero, and the higher order terms are assumed to be small relative to the leading linear terms.

**Figure 3:**
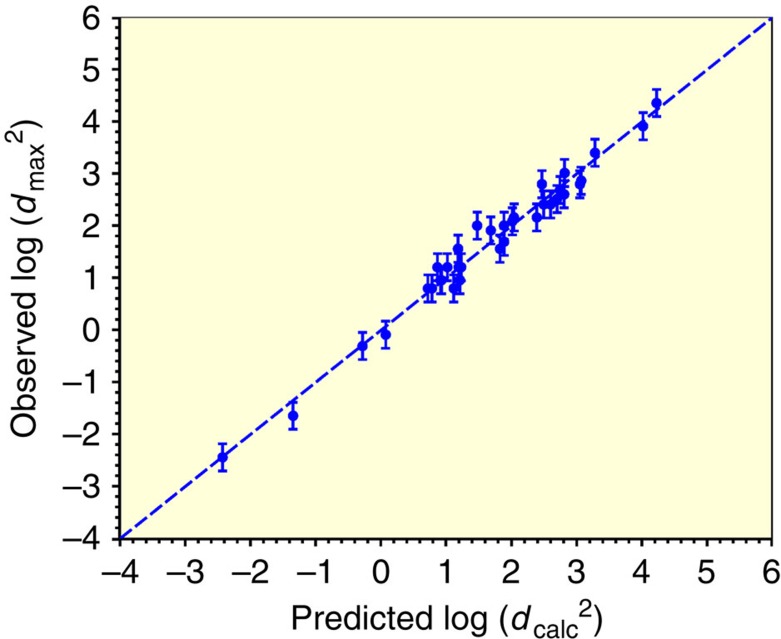
Correlation between observed log (*d*_max_^2^) and predicted log (*d*_calc_^2^). Values of log(*d*_calc_^2^) are obtained from *t*_rg_ and *m* using equation (2). Dashed line is the result of a linear regression least squares fit with fitting parameters 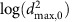
=−10.36, *Λ*_t_=25.6 and *Λ*_m_=0.0481. The coefficient of determination for the linear fit is *R*^2^ is 0.980. Error bars given by equation (3) in text.

Fitting the data in Supplementary Table I using equation (2) and retaining only the linear terms in the Taylor series, we obtain best values for the fitting parameters 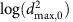
=−10.36, *Λ*_t_=25.6 and *Λ*_m_=−0.0481. The quality of the fit is displayed by plotting the experimental data for log(*d*_max_^2^) versus the optimized prediction, log(*d*_calc_^*2*^), of equation (2) as shown in Fig. 3. The coefficient of determination for this fit is *R*^2^=0.980. Equation (2) accounts for a remarkable ∼98% of the variance in log(*d*_max_^2^). Using both *t*_rg_ and *m* versus either alone increases *R*^2^ from ∼0.46/0.60 to 0.980. This statistically compelling result establishes the relevance of both parameters and yields a useful quantitative prediction of GFA. Experimental uncertainties in *d*_max_, *t*_rg_ and *m* can be estimated. The errors in these values are taken to be random and will contribute to the observed misfit between the model prediction of equation (2) and the experimental GFA data. Uncertainties of *σ*_*d*max_/*d*_max_∼0.15, *σ*_t_∼0.006 and *σ*_*m*_∼3 are estimated to be representative errors in the experimental determination of *d*_max_, *t*_rg_ and *m*, respectively, for the present database. The basis for these error estimates is discussed in the Supplementary Information. An analysis of the variance *σ*^2^ for the misfit between log(*d*_calc_^2^) and log(*d*_max_^2^) contributed by these estimated experimental errors yields:


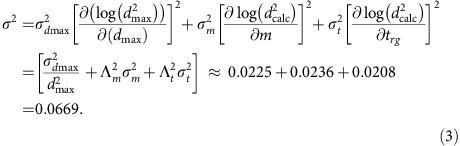


The estimated experimental errors yield a s.e. of *σ*≈0.26 for the misfit between the experimental log(*d*_max_^2^) and the predicted log(*d*_calc_^2^). The error bars displayed in Fig. 3 were chosen to have this value.

## Discussion

The actual variance of the misfit in Fig. 3 gives a s.d. of ∼0.23, very close to that expected from experimental error alone (0.26). Adding a third input parameter such as *γ*_XL_ to the expression for GFA will not be useful since experimental errors will mask any improvement in the prediction of GFA versus the simple two-parameter model of equation (2). In other words, the prediction of a better model could not be empirically distinguished from that of equation (2).

From a modelling perspective, we have assumed *t*_rg_ and *m* to be uncorrelated independent variables. Analysing an *m* versus *t*_*rg*_ plot for our database can test this assumption. A weak anti-correlation (alloys with higher *t*_rg_ have statistically slightly lower *m*) with corresponding coefficient of determination of *R*^2^∼0.08 is obtained from such a plot. For the present database, *t*_rg_ and *m* are apparently very weakly correlated, if at all. One consequence of a weak anti-correlation will be a small systematic over-estimate of the magnitude of Λ–parameters obtained in Figs 1 and 2 versus those obtained from the bilinear fit of Fig. 3. For the bilinear fit, we obtained *Λ*_t_≈25.6 and *Λ*_m_≈=0.0481. For the single parameter analysis of Figs 1 and 2, we obtained *Λ*_t_≈28.5 and *Λ*_m_≈−0.057.

In summary, we have demonstrated that knowledge of *t*_rg_ and *m* is sufficient to predict GFA within experimental uncertainties in the relevant variables. This result is unexpected and must be rationalized in the context of traditional nucleation theory. We proceed to discuss the implications of this result and to examine the practical utility of equation (2).

**Figure 4:**
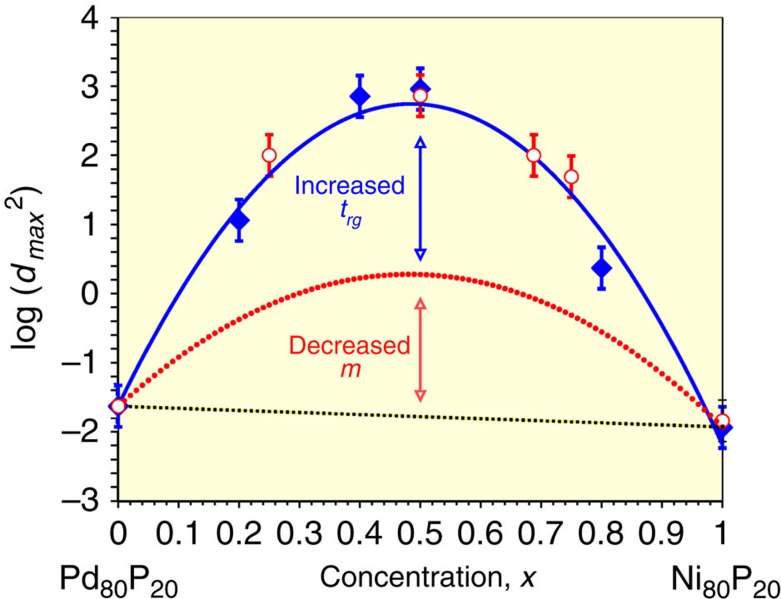
GFA versus composition diagram for the pseudobinary (Pd_1−*x*_Ni_*x*_) _80_P_20_ system. Input data are taken from refs 25, 26, 27, 28, 29, 30, 31, 32. Solid blue square symbols are calculated GFA from experimental *t*_rg_ and *m* values^31^,^32^ using equation (2). The solid blue line is a fit to the calculated GFA values. Dotted red line is a parabolic fit to the calculated contribution from fragility. Red open circles are experimental GFA data from refs 25, 26, 27, 28, 29, 30. The reader is referred to the Supplementary Information for details. Error bars represent experimental uncertainty of 15% in the maximum casting diameter as discussed in text.

Perhaps the most practical result of the present work lies in clarifying the relative roles played by Turnbull's parameter and Angel's fragility concept in achieving very high (*d*_max_>1 cm) GFA. While bulk glass formation has commonly been associated with low melting eutectic alloys, it is the combination of a deep eutectic with strong-liquid rheology that underlies superior glass-forming ability. This is best illustrated by quantifying the relative contributions of *t*_rg_ and *m* to the GFA of superior eutectic glass formers. As an example, consider the Pd–Ni(Cu)–P alloy system. This system includes a ternary Pd–Ni–P bulk glass forming alloy with *d*_max_∼3 cm (see Supplementary Table I) and a related quaternary alloy of Pd–Ni–Cu–P with observed *d*_max_>8 cm (refs 25, 26, 27, 28, 29, 30). The simple binary eutectic alloys Ni_81_P_19_ and Pd_81_P_19_ (*T*_L_=1,143 and 1,044 K, respectively) form rapidly quenched glassy ribbons of thickness ∼50 μm. The equivalent rod diameter is *d*_max_∼140 μm (Supplementary Information). Ternary alloys along the (Pd_1−*x*_Ni_*x*_)_80_P_20_ composition line comprise a pseudobinary eutectic system forming bulk glass rods at *x*=0.5 having an observed *d*_max_>25 mm (refs 25, 26). As a function of composition *x*, *d*_max_^2^ exhibits a broad maximum that varies by 4–5 orders of magnitude along the pseudobinary line^25^,^26^,^28^,^30^. Chen^31^,^32^ carried out thermal characterization and systematic creep studies for glasses along this line and reported highly reliable viscosity data well above *T*_g_. Accurate *m* values can be obtained using his Vogel-Fulcher-Tamann viscosity fits (see Supplementary Table I and discussion in Supplementary Methods). Using equation (2), one may separate the enhancement of GFA from increasing *t*_rg_ from that attributable to decreasing *m*. The logarithmic form of equation (2) means that the variation of *d*_max_^2^ with *x* is a product of two factors, attributable respectively to the variation in *t*_rg_ and *m* (see Supplementary Methods and Supplementary Figs 3-5 for details of the *t*_rg_ and *m* variation with *x*). Figure 4 illustrates this separation of factors and demonstrates that *t*_rg_ and *m* play roughly comparable roles in maximizing log(*d*_max_^2^) at *x*=0.5 as one traverses the pseudobinary series. The nucleation nose time *τ*_X_* of the ternary (Pd_0.5_Ni_0.5_)_80_P_20_ exceeds that of either binary alloy by over four orders of magnitude! Two orders of magnitude are attributable to an increase in *t*_rg_, while another two orders of magnitude arise from a decrease in *m*. Interestingly, the addition of Cu to the ternary alloy (see entry No. 26 in Supplementary Table I) to obtain the precise quaternary eutectic alloy Pd_42.5_Cu_30_Ni_7.5_P_20_ increases *d*_max_^*2*^ by an additional factor of ∼15 yielding the best glass forming alloy known. The quaternary eutectic alloy actually has a larger *m* (58 versus 48) than the ternary, but a substantially higher *t*_rg_ (0.679 versus 0.589) that more than offsets the higher fragility and is responsible for the elevated GFA of the quaternary eutectic versus the ternary alloy. By applying such analysis, it can be generally shown that superior glass formers (for example, Vitreloy 1 with *d*_max_∼4–5 cm, or Mg–Gd–Ag–Cu-Al with *d*_max_∼3 cm), owe their elevated GFA (compared with the simpler binary or ternary basis alloys) to a confluence of two roughly comparable multiplicative factors arising from low-lying eutectic melting and strong-liquid rheology, respectively. This type of analysis provides a quantitative means to rationalize the underlying factors responsible for the variation of GFA in complex multicomponent systems.

The success of equation (2) in predicting GFA over a broad range of metallic alloys would not be possible if heterogeneous nucleation effects played a substantial role in limiting experimentally measured GFA. Heterogeneous nucleants such as oxide inclusions ought to exhibit widely varying catalytic potency that depends on their size, crystal structure and effectiveness as a template for nucleation of each competing crystalline phase. Large variations in catalytic activity would mask any systematic dependence of intrinsic GFA on *t*_rg_ and *m*. Apparently, common experimental measures used to suppress heterogeneous nucleation are relatively effective. These measures include: (1) using high purity starting materials^11^,^25^, (2) melt overheating above the oxide phase liquidus temperature to dissolve oxide inclusions in the liquid^33^,^34^,^35^,^36^,^37^,^38^,^39^,^40^, (3) use of non-crystalline containers^11^, (4) container-less processing combined with overheating^33^,^34^,^35^,^36^,^37^,^38^,^39^ and (5) fluxing methods to remove oxide inclusions^11^,^20^,^21^,^25^,^26^,^40^,^41^. Apparently, these measures are effective in achieving near homogeneous nucleation conditions during melt undercooling and glass formation.

The absence of the liquid/crystal interfacial energy in equation (2) is unexpected and raises fundamental questions. Assuming that the crystal nucleation rate controls GFA, then what determines the nucleation barrier Δ*G*(T*) in equation (1) at temperature T*? Our result might be understood if ΔG(T*) were uniquely determined by *t*_rg_ and *m*. In the context of CNT, this might imply that *γ*_XL_ is some unique function of *t*_rg_ and *m* and thus already implicitly included in equation (2). This seems somewhat implausible since *γ*_XL_ should depend on the crystal structure of the nucleating phase, its composition (and that of the parent liquid), temperature and so on. Alternatively, if transient nucleation is important, then the incubation time to establish a steady state population of crystalline embryos may control GFA. This time might be determined by *t*_rg_ and *m*. The incubation time should be related to the time required for development of local chemical fluctuations on a spatial scale comparable to that of the critical nucleus. For instance, the early work of Borelius *et al.*^42^ suggests that the free energy cost of chemical fluctuations in multicomponent alloys might dominate the topological contribution to the nucleation barrier. Other recent work by Desre *et al.*^43^,^44^ and the work of Wu (refs 45, 46) emphasize the role of composition fluctuations in crystal nucleation for multicomponent liquids. A nucleation rate limited by such chemical fluctuations might explain the success of equation (2). However, there remains unanswered the question of what determines the relevant spatial scale of the critical fluctuation, that is, the critical nucleus size?

The transformation from a liquid to a fully crystallized solid generally involves both the crystal nucleation rate and crystal growth velocity^47^,^48^. A sluggish nucleation process is a sufficient, but not necessary condition for glass formation. Orava and Greer^49^ have argued that sluggish crystal growth is the likely rate-limiting factor that controls the apparent GFA of many silicates and other molecular glasses. As noted by Greer^50^, growth velocities are independent of *γ*_XL_. Thus, growth controlled crystallization would explain the absence of a role played by interfacial energy in determining GFA. However, the analysis of ref. 49 included only one metallic system, a low-melting Cu_50_Zr_50_ alloy with *t*_rg_=0.55, *m*=58, *d*_max_=2 mm and corresponding *τ*_X_*∼0.04 s (Supplementary Table I). This alloy crystallizes to a single crystalline phase having the same composition as the parent liquid, that is, by polymorphic crystallization, not typical of most metallic glass formers. Recent measurements of the multiphase eutectic growth velocity near T* in several easy glass formers by time-resolved infrared imaging^51^ give relatively high velocities ranging from several cm s^−1^ for Zr-based bulk glasses up to several m s^−1^ for high-GFA eutectic Ni-alloys (see ref. 51 and also J.P. Schramm, G. Kaltenboeck, M.D. Demetriou, and W.L. Johnson, manuscript in preparation). Such high-growth velocities indicate that the observed GFA of these eutectic glass formers must be determined by nucleation rates. The case of elemental metals alluded to in the introduction^1^,^2^ is of interest in this context. Zhong *et al.*^1^ experimentally demonstrated that cooling rates of 10^14^ K s^−1^ are sufficient to suppress crystal growth and produce glass during rapid transient melting of crystalline Ta and V nano-bridges. In this case, the crystalline phase is present, but cannot regrow due to ultra-rapid transient cooling. By contrast, An *et al.*^2^, showed that lower cooling rates of ∼10^12^ K s^−1^ are sufficient to form glass in simulations where nano-droplets of liquid Cu are quenched onto an amorphous substrate (with no crystalline nucleus present). Further investigation is clearly required to clarify the relative roles of nucleation versus growth in specific cases.

The glass-forming ability of metallic alloys is alternatively defined by either the crystallization nose time of the TTT diagram or by a critical casting thickness for avoiding detectable crystallinity. We have compiled and critically assessed available data for a diverse set of ∼40 of metallic glass-forming alloy systems where reliable experimental GFA, liquid rheology and thermodynamic data are available. This database is used to develop a universal expression that quantitatively predicts GFA based only on Turnbull's parameter *t*_*rg*_ and Angell's liquid fragility parameter *m* as independent variables. The analysis yields two fitting parameters *Λ*_t_ and *Λ*_m_ characterizing the intrinsic variation of GFA with *t*_rg_ and *m*, respectively. The expression quantitatively predicts the critical casting thickness or nucleation nose time for forming glassy alloys with the uncertainty expected from experimental errors in the relevant parameters alone. We interpret this universal GFA expression as a description of the crystal nucleation rate in the undercooled liquid state. Evidence suggests that for typical multicomponent eutectic glass formers, crystal growth velocities (specifically eutectic growth velocities) at temperatures near T^*^ are sufficiently high to justify neglecting the influence of sluggish growth in limiting observed GFA. From the present results, one may conclude that:

The conditions for homogeneous nucleation must be generally approached in experimental studies where common practices are employed to suppress heterogeneous nucleation. The crystal-liquid interfacial energy does not explicitly appear to play a significant role in determining GFA. A relatively simple theory of glass formation and crystallization ought to be possible for metallic systems. Traditional nucleation theory offers no obvious explanation for why this turns out to be the case.

## Methods

### Metallic glass database construction

The present article is based on a broad survey and critical assessment of published experimental data on metallic glasses. This assessed data have been compiled into a metallic glass database that is presented as Supplementary Table I. The database includes selected data for 42 separate alloys. The compiled data includes values of the rheological glass transition temperature *T*_g_, alloy liquidus temperature *T*_L_, reduced glass transition temperature *t*_rg_=*T*_g_/*T*_L_, Angell fragility parameter *m*, critical casting diameter *d*_max_ of a metallic glass rod, the calculated critical casting thickness *d*_calc_ based on equation (2) in the text, the estimated nucleation nose time *τ*_X_*_est_ based on a scaling relation with *d*_max_, and an experimentally measured nucleation nose time *τ*_X_*_TTT_ obtained directly from a measured TTT diagram. The detailed assessment of all parameters is described in detail in the Supplementary Information.

The selection of the alloy entries in the metallic glass database was determined by the availability of consistent and reliable data for the various parameters. For instance, values of *T*_g_ in the database are based on the rheological definition of the glass transition, *η*(*T*_g_)=10^12^ Pa-s. This requires the availability of equilibrium liquid viscosity data in the vicinity of the glass transition. Determination of Angell's *m* parameter requires accurate equilibrium viscosity data over a typical range from 10^8^ to 10^13^ Pa s^−1^ surrounding the glass transition. Determination of *d*_max_ for a given alloy requires systematic and controlled quenching experiments. Direct determination of *τ*_X_* requires a measured experimental TTT diagram. Such diagrams are available only for a limited number of alloy systems that exhibit sufficiently large *d*_max._ Based on this limited number of systems, an empirical relationship is established between *τ*_X_* and *d*_max_.

The Supplementary Information includes discusses the methods and criteria used to assess each parameter in the database. In addition, uncertainty in each parameter arising from experimental error and the propagation of experimental errors in the determination of the parameter are discussed. The estimated errors in the parameters have been used in the main article to assess the statistical significance of the correlations developed in the paper. For example, the reader is referred to the Supplementary Information for a discussion of the analysis of variance presented in equation (3) of the main article.

## Additional information

**How to cite this article:** Johnson, W. L. *et al.* Quantifying the origin of metallic glass formation. *Nat. Commun.* 7:10313 doi: 10.1038/ncomms10313 (2016).

## Supplementary Material

Supplementary Information Supplementary Figures 1-5, Supplementary Table 1, Supplementary Methods and Supplementary References
