# Supplementary material for: Quantifying the origin of metallic glass formation
Source: Nat Commun. 2016 Jan 20;7:10313. doi: 10.1038/ncomms10313 (PMC4735709; doi:10.1038/ncomms10313)
Supplement: Supplementary Information — Supplementary Figures 1-5, Supplementary Table 1, Supplementary Methods and Supplementary References [file ncomms10313-s1.pdf]

Supplementary Information

Supplementary Figures

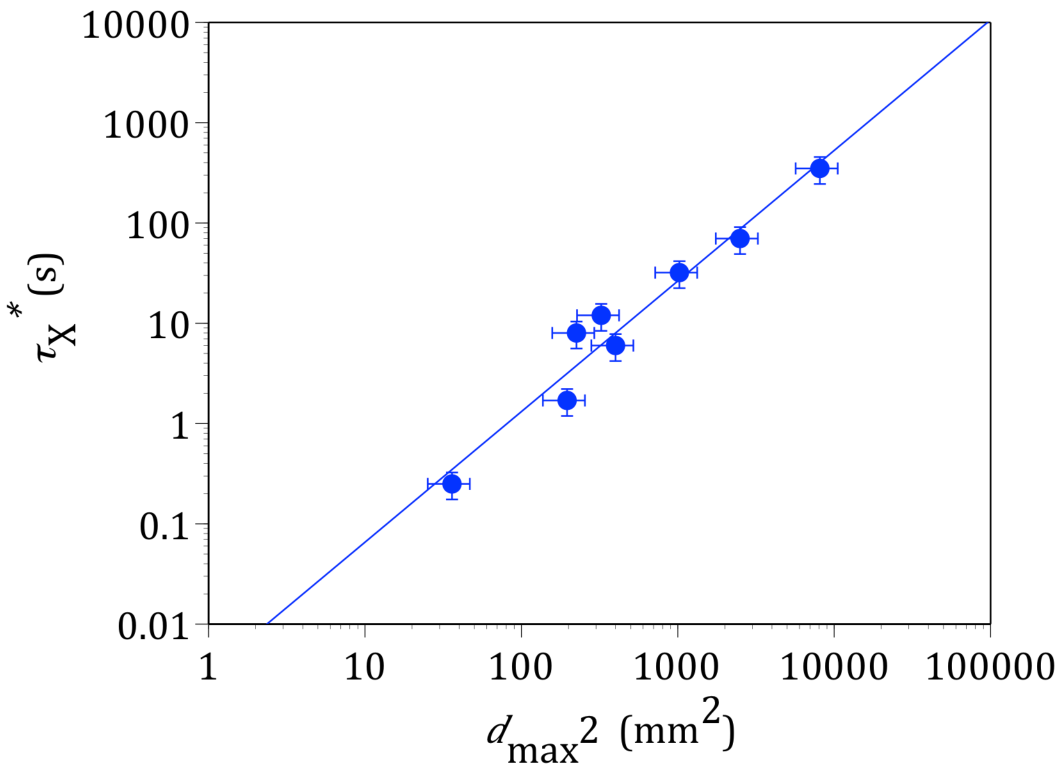

Supplementary Fig.1

Log-Log plot of τ_X^* as a function of d_{max}^2 for eight glass forming alloys for which both quantities have been experimentally measured on the same alloy. The values of τ_X^* for seven alloys were obtained from container-less HVESL experiments where the TTT-diagram was directly measured for a ~2.5 mm diameter liquid drop under near isothermal conditions. One data point (the lowest value of τ_X^* in the figure) is for $Au_{49}Cu_{5.5}Ag_{2.3}Pd_{26.9}Si_{16.3}$ and was obtained by ultrafast calorimetry [see ref.84 for details]. The solid line is a power-law fit as given by Eq. (5) of the Supplementary Methods below. Data used in the figure are listed in Supplementary Table I. The horizontal error bars reflect an estimated error in d_{max} of 15% as discussed in the Supplemental Methods section below. The vertical error bars reflect estimated uncertainty in determining τ_X^* from HVESL experiments.

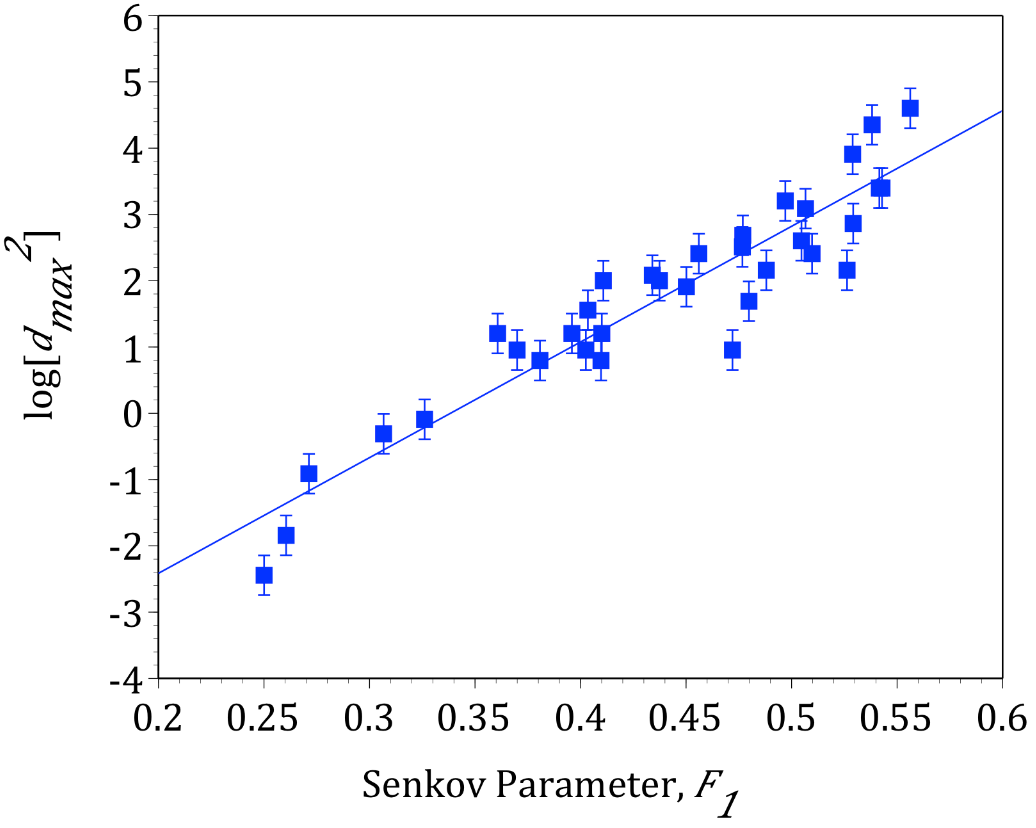

Supplementary Fig. 2

Plot of log(d_{max}^2) as a function of Senkov's F1 parameter as given in eqn. (7) of the Supplementary Methods below. Data used to determine the F1 parameter are given in Supplementary Table I.. A linear least squares fit as shown by solid line which gives a slope of 17.45 and fitting correlation of R^2 = 0.879. Vertical error bars represent and experimental uncertainty in d_{max} of 15% as discussed in the Supplemental Methods below.

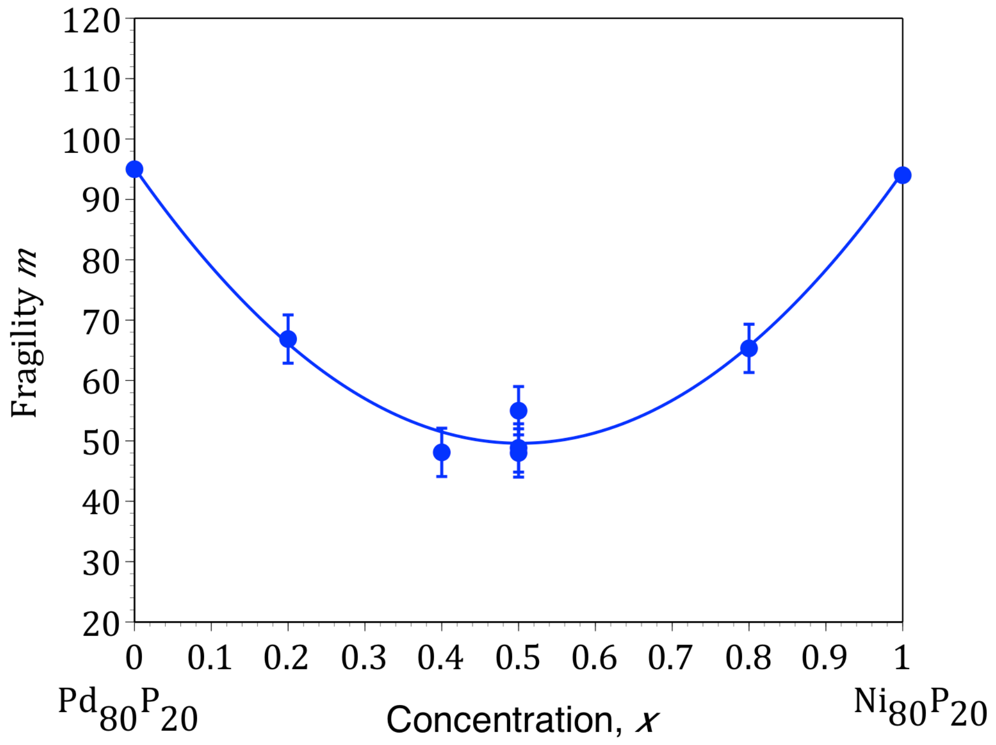

Supplementary Fig.3

Variation of the Angell fragility parameter *m* with the atomic fraction, x, of Ni in ternary alloys of composition $(Pd_{1-x},Ni_x)_{80}P_{20}$. See Supplementary Table I, entries 20-23 and the Supplementary Methods section below for information regarding the data and methods used to construct the plot. The solid curve is a parabolic fit to the composition dependence of the fragility $m(x)$ similar to that used by Chen [see ref. 22] to characterize the variation of the VFT parameters with composition.

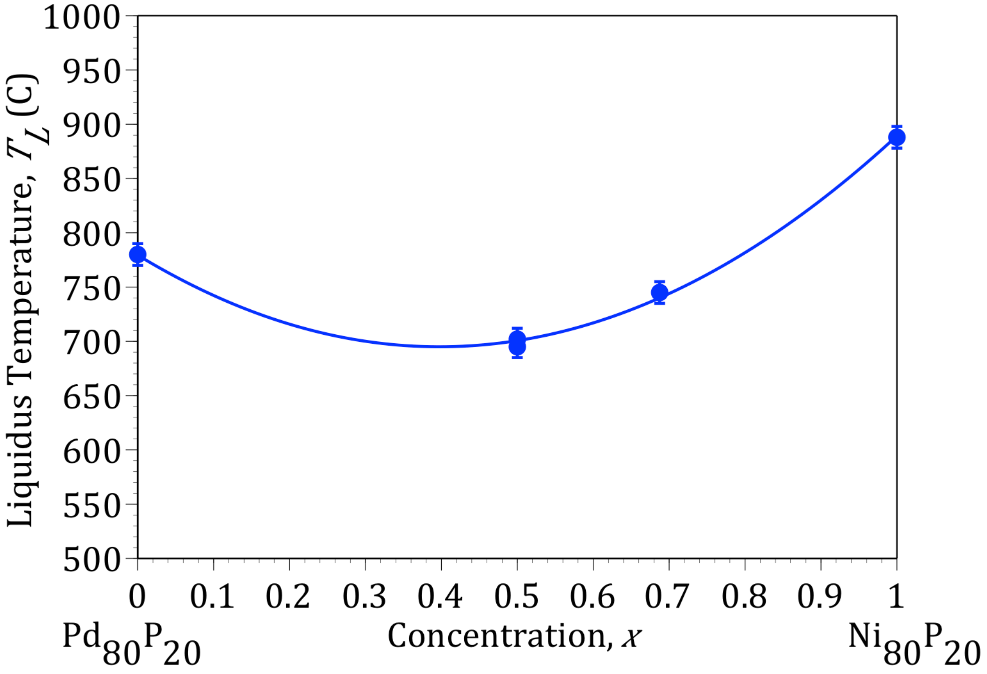

Supplementary Fig.4

Variation of alloy liquidus temperature T_L with the atomic fraction, x , of Ni in the ternary $(Pd_{1-x}Ni_x)_{80}P_{20}$ alloys. See Supplementary Table I, entries 20-23 and the Supplementary Methods section below for a detailed description of the data used to construct the plot. The solid curve is a parabolic fit to the data similar to that used by Chen [22,23] to describe the composition variation of the VFT parameters in the ternary system.

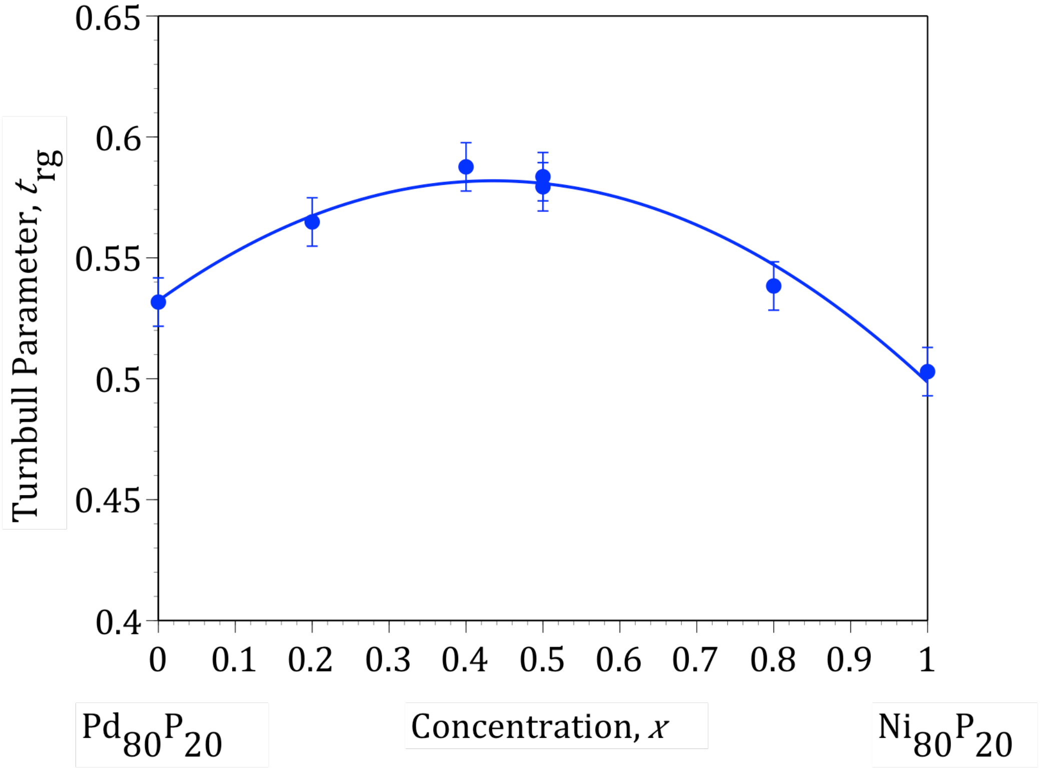

Supplementary Fig.5

Variation of the Turnbull parameter t_{rg} with x, the atomic fraction of Ni in the ternary $(Pd_{1-x}Ni_x)P_{20}$ glass forming alloys. See Supplementary Table I, entries 20-23, and the Supplemental Methods section below for description of the data. Solid curve is a parabolic fit to the data. See Supplementary Methods section below for a discussion of the data.

Supplementary Table I: Metallic Glass Database

A compiled database for 42 metallic glass forming alloys. A description of the entries in the database and the methods used assess the data compiled are provided in the accompanying Supplementary Methods text below. References used as sources for data (last column in the table) are included in the list of references for the Supplementary Information.

**Alloy Systems**<br><br>***Ni-based glasses***	T_g (K)	T_L(K)	t_{rg}	m	d_{exp} (mm)	d_{calc} (mm)	τ^*_{est} (s)	τ^*_{TTT} (s)	References Comments
**1. Ni_{69} $Cr_{8.5}Nb_3P_{19.5}$**	660	1136	**0.581**	**100#**	1	0.9	4.2×10^{-3}	-	[1]
**2. Ni_{69} $Cr_{8.5}Nb_3P_{18}B_{1.5}$**	664	1134	**0.586**	**77**	3	3.5	6.8×10^{-2}	-	[1]
**3. Ni_{69} $Cr_{8.5}Nb_3P_{16.5}B_3$**	668	1134	**0.589**	**59**	10	9.4	1.45	-	[1]
**4. Ni_{69} $Cr_{8.5}Nb_3P_{15.5}B_4$**	668	1187	**0.563**		7		0.59		[1]
**5. Ni_{69} $Cr_{8.5}Nb_3P_{14.5}B_5$**	668	1214	**0.550**		5		0.25		[1]
**6. Ni_{69} $Cr_{8.5}Nb_3P_{13.5}B_6$**	671	1243	**0.540**	**54**	4	2.7	0.14	-	[1]

**7. $Ni_{80}P_{20}$ ribbons**	581	1161	**<u>0.50</u>**	**<u>94</u>**<br>[22]	0.12	0.12	1.9 x 10^{-5}		[2,3,22]<br>*m extrapolated from data of Ref.[22] see text*
				108*					*[4] high T viscosity only*
**8. $Ni_{75}Si_8B_{17}$**	818	1419	**<u>0.583</u>**	-	0.8<br>[6]	0.69	2.4 x 10^{-3}	-	[4,5,6]<br>*d_{max} estimated from strip thickness of 0.55 mm* [6]
				121*					*high T viscosity only*<br>[4]

Fe-based glasses	**T_g (K)**	**T_L(K)**	**t_{rg}**	**m**	**d_{exp} (mm)**	**d_{calc} (mm)**	**τ^*_{est} (s)**	**τ^*_{TTT} (s)**	**References Comments**
**9. $Fe_{80}P_{13}C_7$ *ribbons/capillaries***	736	1258	**<u>0.585</u>**	**-**	0.72	0.93	1.8 x 10^{-3}	-	[4,5,6]
				102*					*high T viscosity only*<br>[4]

**10. Metglas 2826 $Fe_{40}Ni_{40}P_{14}B_6$ *ribbons & bulk 1-2 mm rods***	662	1184	**0.559**	**66** [9]	2-2.5 [7,8]<br>CCR= $8x10^3$ K/s [9,10]	2.8	3.3 x 10^{-2}		[7-10]<br>*CCR from drop tower experiments* [9,10]
**11. $Fe_{79}Si_{10}B_{11}$ *ribbons***	818	1419	**0.576**		0.5		7.2 x 10^{-4}		[5]
**12. $Fe_{74.5}Mo_{5.5}P_{12.5}C_5B_{2.5}$**	695	1219	**0.570**	**63** [15]	3	4.4	6.8 x 10^{-2}	-	[11,15]
**13. $Fe_{68}Mo_5Ni_5Cr_2P_{12.5}C_5B_{2.5}$**	688	1213	**0.567**	**62** [15]	6	4.3	0.39	-	[11,15]
**14. $Fe_{48}Cr_{15}Mo_{14}C_{15}B_6Y_2$**	822	1452	**0.566**	**51** [15]	9 [12]	7.2	1.1	-	[12,13,15]
**15. $Fe_{41}Co_7Cr_{15}Mo_{14}C_{15}B_6Y_2$**	820	1407	**0.583**	**43** [15]	16 [12]	17.8	4.8	-	[12-15]

Precious Metal Alloys	**T_g (K)**	**T_L(K)**	**t_{rg}**	**m**	**d_{exp} (mm)**	**d_{calc} (mm)**	**τ^*_{est} (s)**	**τ^*_{TTT} (s)**	**References Comments**
**16. $Au_{77}Si_{9.4}Ge_{13.6}$**	293	625	**0.469**	**84.7**<br>[16]	0.06	0.05	3.3×10^{-6}		[16,17]
**17. $Pd_{82}Si_{18}$**	657	1071<br><br>1082<br>[19]	**0.613**	**-**	2-3<br>[19]<br><br>~8<br>[18]		4.3×10^{-2}	-	[18-20]<br><br>*high T boron-oxide fluxed spherical ingot up to 8mm diameter reported*
				90*					[4] *from high T viscosity data only*
**18. $Pd_{77.5}Cu_6Si_{16.5}$**	622<br>[22]<br><br>623<br>[21]	1037<br><br>1029<br>[21]<br>[22]	**0.601**	**63**<br><br>63<br>[20]<br><br>61<br>[21]	11<br>[25]<br><br>11<br>[26]<br><br>600 K/s<br>[26]	8	1.9	-	[20-26]<br><br>*estimated critical cooling rate* [26]

**19. $Pd_{79.5}Au_4Si_{16.5}$**	620	1040	0.596	**77**	2	4.2	2.4 x 10^{-2}		[21,22]
**20. $Pd_{64}Ni_{16}P_{20}$**	582	983	**0.571**<br>[22]<br>[30]	**66.9**<br>[22]	4.0<br>[27]<br>[28]	4.1	0.14		[22,27- 30]<br>*see discussion in SI and Fig.4 of main text*
**21. $Pd_{48}Ni_{32}P_{20}$**	565	923	**0.612**<br>[22]<br>[30]	**48.1**<br>[22]<br>[32]		31	26		[22,27-30, 32]<br>*see discussion in SI and Fig.4 of main text*
**22. $Pd_{40}Ni_{40}P_{20}$**	567	963	**0.589**	**48**<br>[22]<br>[27]	25<br>[27,28]	25	15		[22, 27-33,37,38]<br>*see discussion in SI and Fig.4 of main text*
**23. $Pd_{16}Ni_{64}P_{20}$**	567	1048	**0.541**	**65.3**<br>[22]	2.5<br>[27]<br>[28]	2.3	4.3 x 10^{-2}		[22,27-30]<br>*see discussion in SI and Fig.4 of main text*
**24. $Pd_{40}Ni_{40}P_{19}Si_1$**	567<br>[37]	884	**0.641**<br>[37]	**48.2**<br>[37]					[32,37]

**25. $Pd_{40}Cu_{30}Ni_{10}P_{20}$**	578	862	**0.670**	**57.6**<br><br>55.8<br>[24]<br><br>59.4<br>[31]	85<br><br>[40]	107	330	400<br>[36]	[24,31,32,35, 36-40]]<br><br>*reduced nose temp.*<br>*$t^* = T^*/T_L$ =683/862= 0.793* [see ref. 37]
**26. $Pd_{42.5}Cu_{30}Ni_{7.5}P_{20}$**<br>*precise eutectic composition [ref. 31]*	566<br><br>[38]<br>[42]	834<br><br>[38]<br>[41]	**0.679**	**58**<br><br>[42]<br><br>58.5<br><br>[39]	150**<br><br>>80<br><br>[40]<br>[41]	130	1400		[38, 40-42]<br><br>*** estimated from CCR of 0.067 K/s [41]*<br><br>*80mm diameter glass rod fabricated [40]*<br>*see discussion in main text*
Zr- and Ti-based alloys	**T_g (K)**	**T_L(K)**	**t_{rg}**	**m**	**d_{exp} (mm)**	**d_{calc} (mm)**	**τ^*_{est} (s)**	**τ^*_{TTT} (s)**	**References Comments**
**27. $Zr_{50}Cu_{50}$**	673	1208<br>[45]	**0.557**	**58**<br>[44]	2.5	3.0	4.3 x 10^{-2}		[44-46]<br><br>*m evaluated directly from data in [44]*

**28. $Zr_{11}Ti_{34}Cu_{47}Ni_8$**<br>**Vit. 101**	671<br>[49]<br>668<br>[54]	1160<br>[47]<br>[49]<br>[53]	**0.578**<br><br>0.575<br>[54]	**67**<br>[49]<br>59<br>[54]	4<br>[47]	4.7	0.14		[47-49,52-54]<br>*best m from combined low T and high T data*
**29.**<br>**$Zr_{52.5}Cu_{17.9}Ni_{14.6}Al_{10}Ti_5$**<br>**Vitreloy 105**	661<br>[54]	1091<br>1125<br>[54]	0.606	**51.4**<br>[54]	18	22	6.5	12<br>[50]	[50,53,54,56]<br><br>*τ_{ESL} @ 250 ppm oxygen [50]*
**30.**<br>**$Zr_{57}Nb_5Cu_{15.4}Ni_{12.6}.Al_{10}$**<br>**Vitreloy 106**	674<br><br>673<br>[54]	1115<br>[48]	**0.604**	**48**<br><br>50.3<br>47.5	20	24.6	8.4	6<br>[48]<br>[49]<br>[57]	[51-54,56,57]<br><br>*$t^* = T^*/T_L = 0.803$ from [47,48]*
**31. $Zr_{58.5}Nb_{2.8}Cu_{15.6}Ni_{12.8}$**<br>**$Al_{10.3}$**<br>**Vitreloy 106a**	666<br>[55]<br><br>661<br>[54]	1101<br>[55]<br>[51]	**0.605**	**47.5**<br>[56]<br>46<br>[54]	32<br><br>*TTT-curve*<br>[51]<br>[55]	25	28	32<br>[55]	[31,51,54-56]<br><br>*$t^* = T^*/T_L =$ 0.815 from [55]*<br><br>*d_{expt} estimated from τ^*_{ESL} [55]*

**32. $Zr_{60}Cu_{40}Al_{10}$**	706	1123 [59]	**0.619**	**57** [58]	22 [60]	25	10.8			[58-60]
**33. $Zr_{46.75}Ti_{8.25}Cu_{8.25}Ni_{10}Be_{275}$ Vitreloy 4**	595 [67]	1050 [67]	**<u>0.567</u>**	**<u>43.9</u>** [64] [67] [68]	12	10.5	2.3	-		[34,64-68]
**34. $Zr_{55}Co_{25}Al_{20}$**	753 [56]	1293 [56]	**<u>0.582</u>**	**<u>64.5</u>** 69 *ht* 60 *lt* [56]	10 [56-57]	7.3	1.5	1.7 [56] [57]		[56,57] *$t^*= T^*/T_L$ = 980/1293=0.758 [56,57]*
**35. $Zr_{41.2}Ti_{13.8}Cu_{12.5}Ni_{10}Be_{22.5}$ Vitreloy 1**	608 613 [31] [61] [67]	991 [31] [61] [63]	**<u>0.614</u>**	**<u>43</u>** 42 44.4 [31] [68] [54]	50 [62] 40** [63]	42	50	70 [56] [57] [63]		[31,34,54,56,57,61-63,65,67,68] *preferred m from digitized data* *$t^*= T^*/T_L$ = 0.804 [56,57,63]* ***d_{max} from TTT-diagram estimated to be ~40mm*

Mg- & La-based alloys	**T_g (K)**	**T_L(K)**	**t_{rg}**	**m**	**d_{exp} (mm)**	**d_{calc} (mm)**	**τ^*_{est} (s)**	**τ^*_{TTT} (s)**	**References Comments**
**36. $Mg_{65}Cu_{25}Y_{10}$**	413<br>420 [72]	735<br>739 [72]	**0.562**	**44.5**<br>44.5 [60]<br>47.1 [27]	7	8.8	0.6		[31,60,69-72]
**37. $Mg_{59.4}Cu_{23}Ag_{6.6}Gd_{11}$**	425 [72]	700 [72]	**0.607**	**44**	34	27	33	-	[60,70-72]
**38. $Mg_{61}Cu_{28}Gd_{11}$**	418 [72]<br>422 [70]	734 [72]<br>737 [70]	**0.569**	**38.1** [70]<br>47 [72]	12 [70]	9.3	2.3		[70-72]
**39. $La_{55}Al_{25}Ni_{20}$**	447	876	**0.510**	**37.3**<br>35 [73]<br>39.5 [31]	3	3.4	6.8 x 10^{-2}		[31,60,73,75]

**40. $La_{62:5}Al_{12:5}Cu_{15}Ni_5Ag_5$**	391	717	**0.545**		8		0.82		[72,74,75]
**41. $La_{62.5}Al_{12.5}Cu_{10}Ni_5Co_5Ag_5$**	404	691	**0.585**		12		2.3		[72,74,75]
**42. $La_{65}Al_{14}Cu_{9.2}Ag_{1.8}Ni_5Co_5$**	419	687	**0.610**		30		24		[76]

Footnotes for Supplementary Table I

Values extrapolated using the composition dependence of m (measured data for 1.5-6 at.% boron are extrapolated to 0 % boron) using fitting function in ref.[1]

* These values of *m* are estimated from high temperature data only and based on the early studies of equilibrium liquid viscosity as reported by Davies [4]. These data were not used in Figs. 2 and 3 since such values of m are known to be overestimated compared with those based on low temperature viscosity data. See discussion in SI text.

In general, when low temperature viscosity data are reported in terms of VFT fitting parameters (D and T_0), the Angell *m* parameter has been analytically determined from the identity relation:

$$m= [DT_0T_g]/[ln(10)(T_g-T_0)^2]$$

Supplementary Methods

Methods for Compiling the Metallic Glass Database

The data used to compile Supplementary Table I are taken from the published literature on metallic glasses covering the time frame from 1960 to the present. Each entry in the table represents a single alloy composition. In some cases, several entries of different compositions within the same alloy system are included to illustrate the composition variation of glass formation and alloy properties in a given system. Column 1 gives the alloy composition in atomic percentages. The second column gives the rheological glass transition temperature, T_g (K) defined as the temperature at which the equilibrium liquid viscosity is 10^{12} Pa-s. Column 3 gives the alloy liquidus temperature, T_L (K) defined as the temperature above which the equilibrium alloy is fully liquid (contains no solid phases). Column 4 gives the dimensionless Turnbull parameter, $t_{rg} = T_g/T_L$ The value of t_{rg} in bold print is the preferred value used for GFA analysis in the paper. Note that in the literature, this is often labeled T_{rg}. We prefer the lower case letter to indicate the dimensionless nature of this ratio. Column 5 gives the dimensionless Angell Fragility Parameter m, defined as:

$$m = d(log\eta)d(T_g/T)/_{Tg/T = 1} \qquad (1)$$

where η is the temperature dependent viscosity in units of Pa-s and T_g is the rheological glass transition temperature. Values of m in bold print are the preferred values used for analysis in the paper. Column 6 is the experimentally observed glass forming ability of the alloy expressed in terms of the maximum reported diameter of a rod, d_{max}, (in mm) for which the liquid alloy can be quenched to a glass with no detectable crystallinity. Column 7 is the predicted glass forming ability obtained from equation (3) in the main text. Column 8 expresses GFA in terms of an equivalent nose time, τ_X^* (in s). This nose time is defined by onset of recalescence in a spherical droplet ~2.5 mm in diameter (typical used in High Vacuum Electrostatic Levitation, HVESL, processing experiments) under near isothermal conditions at the

respective T^* of the sample. This is estimated from the experimental d_{max} values using an empirical scaling relationship $\tau_X^* \sim \alpha\, d_{max}^{n}$ with α and the exponent n are determined by a best fit to data where both τ_X^* and d_{max} have been experimentally determined for the same alloy (see discussion and eqn.(5) below). Column 9 gives the experimental values of τ_X^* taken directly from a measured TTT-diagram obtained using containerless HVESL processing. Such experimental TTT-diagrams are available for a limited number of alloy entries (~10). Column 10 provides a list of references for each alloy entry from which the respective data were taken. Reference numbers are also indicated in other columns to assist the reader in identifying the source of specific parameter values. Column 10 also contains comments regarding the entries. Below, we describe the procedures and methods used to assess the literature data.

A. Assessment of Viscosity data (T_g and m)

Low temperature viscosity data in the literature near and above T_g are obtained using techniques such as Parallel Plate Rheometry [77], Beam Bending [64], and Creep Rate studies on wires and ribbons [21]. Data typically cover the range of viscosity from 10^6-10^{14} Pa-s. The lowest measureable viscosity is generally limited by intervening crystallization of the metallic glass. Data for alloys with poor stability against crystallization (small $\Delta T = T_X - T_g$, where T_X is the crystallization temperature) are often restricted to viscosities above 10^{10} Pa-s. Reported data are most often fit using the Vogel-Fulcher-Tammann (VFT) equation:

$$\ln(\eta/\eta_0)=\exp[DT_0/(T-T_0)] \qquad (2)$$

where D, T_0, and η_0 (the liquid viscosity in the high temperature limit) are fitting parameters [78]. It is frequently assumed that $\eta_0 \approx 10^{-3}$ Pa-s. For most studies, best values of D and T_0 and η_0 are tabulated. As seen in eqn. (1), the Angell Fragility parameter, m, is the slope of a plot of $\log(\eta)$ vs. T_g/T evaluated at T_g [78]. It is easily shown that m can be expressed in terms of the VFT parameters as:

$$m = (DT_0T_g)/[\ln(10)(T\text{-}T_0)^2] \quad (3)$$

Tabulated values of m in Table I were derived from this relationship for cases where the VFT-parameters were reported. Experimental uncertainty in fitting parameters D, T_0, and the rheological T_g are propagated and give a corresponding uncertainty in m. The typical error in determining m is estimated below.

Low temperature viscosity data can also be fit using other model functions such as the Cooperative Shear Model [79]. In this case, the m values are determined from:

$$m = 15(1+2n) \quad (4)$$

where n is an index describing the exponential decay of the flow barrier $W(T)$ vs. T/T_g [79]. Clearly, m can also be obtained directly from the slope of a $\log(\eta)$ vs. T_g/T plot at $T = T_g$. This direct method was used to obtain m in cases where digitized viscosity-temperature data are available. For some entries in the Table, multiple m values from independent reports are available. The scatter in data for the same alloy provides an indication of the uncertainty in m values among various investigators using different techniques.

Viscosity data at high temperature (near and above T_L) are obtained using Liquid Drop Oscillation Decay [56,80]. Here, the viscosity is determined from the exponential damping time of mechanical dipole oscillations (Lame oscillations) of a liquid droplet. The method is limited to relatively low viscosity values (< 100 mPa-s) where such oscillations are under-damped and is useful mainly for liquids above T_L. Capillary Flow Viscometry and Couette (Rotating Cup) Viscometry are other common methods to measure viscosity of relatively fluid liquids (< 10^2 Pa-s). In general, high temperature viscosity data below T_L is limited to temperatures where the undercooled liquid is stable against crystallization on the time scale of the measurement. Reported high temperature viscosity data on metallic glass forming alloys is available for viscosities below about ~1Pa-s. High temperature data is often

fit to the VFT-law by requiring that the VFT fit be constrained to give $\eta(T)$ = 10^{12} Pa-s at a nominal $T = T_g$. Such fits are an interpolation/extrapolation of the viscosity curve by 11 orders of magnitude or more (from ~ 10^0 – 10^{12} Pa-s), a region where little data is available regarding the temperature dependence of $\eta(T)$. Using high temperature data alone to estimate m thus results in large typical errors. It is also generally found that m values obtained from high temperature data alone are consistently larger than those obtained from low temperature viscosity data (in the neighborhood of T_g where m is actually defined). Values of m compiled in Supplementary Table I are in the range $35 < m < 100$. Using high temperature data alone gives values of m as much as ~20 higher than those obtained from low temperature data (e.g. see [56,80]) Such large errors and inconsistencies make these values almost useless in attempts to correlate GFA with fragility. The m values used in our analysis of GFA (the bold-face entries for m in Supplementary Table I) are based on either low temperature data alone, or on a combination of both low and high temperature data.

B. GFA and critical rod diameter d_{max}

Throughout the paper, we have defined GFA in terms of d_{max}, the maximum diameter of a long rod that can be quenched to a glass with no detectable crystallinity. This definition represents a practical approach since data for d_{max} are most widely available. The d_{max} values listed in the Supplementary Table I are maximum rod diameters obtained by, (1) water quenching the melt from above T_L in thin walled quartz tubes (0.5 or 1.0 mm wall thickness), by (2) metal mold casting typically done by pouring or injecting the melt into a cylindrical copper mold, or (3) melt spinning ribbons or wires of varying thickness to determine a maximum ribbon (wire) thickness which can be made amorphous. The figures in the main text display GFA in terms of d_{max}^2. This choice is based on the assumption that $\tau_X^* \sim d_{max}^2$ and the fact that the thermal diffusivity, D_{th}, of all metallic glass forming liquids is roughly the same. Measured values of D_{th} for metallic glass forming liquids fall in range 2 mm^2/s < D_{th} < 5 mm^2/s [81,82]. Consider the transient solution to the Fourier heat

flow equation for quenching a long cylindrical rod at some initial temperature $T > T_L$ quenched to ambient temperature by suddenly clamping the sample surface temperature to that of a stirred water bath (absent any boiling of the water). The leading term in a series for transient temperature distribution $T(r, t)$ has a radial dependence given by the zeroth order Bessel Function $J_0(\lambda_i r)$ with $\lambda_i = 2.405/(d/2)$ and the time dependence ~exp[$-D_{th}\lambda_i^2 t$]). The rod centerline temperature decay is dominated by this term with a relaxation time $\tau_{th} \sim d_{max}^2/(23.14\, D_{th})$. For a typical value of d_{max} = 1 cm and a typical liquid thermal diffusivity of D_{th} = 0.04 cm^2/s, we have τ_{th} ~ 1 s for the decay time of the centerline temperature. To avoid crystallization, one must roughly have $\tau_X^* \approx \tau_{th} = (d_{max}^2)/(23.14\, D_{th})$. This establishes a relationship between, τ_X^* and d_{max}^2, as two alternate measures of GFA. In the above example we find that a d_{max} of 1 cm is equivalent a nose time of $\tau_X^* \sim 1$ s. Referring to Table I, we see that alloys with d_{max} ~ 1 cm (see, for example, entry No.'s 18, 33, 34, and 41 in Supplementary Table I) indeed have τ_X^* values of roughly 1 s. The quantitative nature of the agreement is likely fortuitous given the approximations made. For one thing, the measured values of τ_X^* refer to an HVESL experiment for an isothermal sphere of diameter ~ 2.5 mm (see above). It is not clear how the effective volume of a quenched rod exposed to the greatest nucleation rate (volume exposed to the lowest cooling rate) versus the isothermal sphere (of fixed volume) effect the definition of τ_X^*. Nonetheless, our estimate of τ_{th} verifies that d_{max}^2 and τ_X^* are related. The success of Eq. (2) suggests that d_{max}^2 is indeed a useful definition of glass forming ability. Direct measurements of τ_X^* are actually available for a limited number of cases (Supplementary Table I). Based on those data, we shall establish an empirical scaling relation between τ_X^* and d_{max}^2 as described in the next section. This relation can be used to roughly convert each measure of GFA to the other.

C. Experimental measurements of τ_X^* and empirical correlation with d_{max}^2

To measure $\tau_X(T)$ directly, containerless High Vacuum Electrostatic Levitation (HVESL) experiments have been employed [56,63]. Here, the sample is a

liquid droplet typically 2.5 mm in diameter. The sample is heated to a temperature above T_L, and then allowed to cool by free radiative heat loss (i.e. the Stephan-Boltzmann "T^4" law). Observed free radiative cooling rates are typically ~5-30 K/s scaling roughly as T_L^4. The internal Fourier thermal relaxation time of a 2.5-mm diameter droplet is less than ~0.1 s [83]. The sample cools sufficiently slowly that internal temperature gradients are small within the sample. The sample is near isothermal. A typical experiment to measure $\tau_X(T)$ involves heating the sample well above T_L using power absorbed from a laser beam(s) to achieve an initial steady-state temperature. The laser is suddenly switched off to allow free radiative cooling. Upon reaching some target temperature below T_L, the laser is turned back on at a new input power level chosen to maintain the drop at steady state at the new target temperature. Basically, the drop is free cooled to some temperature lower than T_L, then held there at constant temperature. Crystallization is detected by an abrupt temperature rise associated with recalescence. The elapsed time to recalescence at the target temperature is measured. Repeating the process for various target temperatures around T^* determines the $\tau_X(T)$-curve or TTT-diagram in as direct a manner as possible. At each temperature, the droplet is near isothermal and one measures the waiting time to recalescence (generally quite abrupt and well defined near T^*). Since the volume of the droplet is known, one may express the result as an intensive nucleation rate (nuclei per unit volume per sec.) using observed nose time, τ_X^*. The data for the τ_X^* is generally well defined and reproducible. Unfortunately, such experiments are limited to glass forming alloys where τ_X^* is sufficiently long to permit free radiative cooling of the droplet to T^* within a time scale less than τ_X^*. Practically, this requires $\tau_X^* > \sim 1$ s for a 2.5 mm diameter liquid drop. Thus, HVESL data for τ_X^* are only for high GFA alloys (roughly 10 entries in Table I). Supplementary Fig.1 is a plot of d_{max}^2 versus τ_X^* for 8 alloys where both are measured. The data are plotted on a log-log plot to assess whether power-law scaling $\tau_X^* \sim d^n$ is appropriate. A best least squares fit gives:

$$\tau_X^* = 0.00419\ d_{max}^{2.54} \qquad (5)$$

where τ_X^* is in seconds and d_{max} in millimeters This result is strictly empirical. No effort has been made to interpret the exponent n = 2.54 except to note that the above arguments suggest $n \sim 2$ when the effective volume exposed to the maximum nucleation rate is not considered while one expects $n > 2$ if the effective volume scales with some power of the characteristic sample dimension. In Supplementary Table I (column 8), we have the scaling relation in Eqn. (5) to estimate τ_X^* using d_{max} as input. As such, the values for τ_X^* are estimated equivalent waiting times for a spherical droplet of diameter ~ 2.5 mm under near isothermal conditions at T^*. Normalizing to a unit volume (e.g. 1 m^3) allows one convert this to an estimated intensive normalized nucleation rate of ~ 1.2 x 10^8 ($1/\tau_X^*$) (in units of nuclei/m^3-s) from data in Supplementary Table I. This assumes that a steady state nucleation rate is relevant. This assumption may not be true if, for instance, transient nucleation determines τ_X^*. In that case, τ_X^* may instead represent an incubation time for the onset of nucleation at a much higher steady-state rate.

D. Estimation of experimental errors

Experimental errors in t_{rg}, m, and d_{max} limit the ultimate accuracy achievable when validating Eq. (2) of the article. Experimental error in Turnbull's parameter, $t_{rg} = T_g/T_L$ arises from uncertainty in T_g and T_L. The experimental error in determining T_g is relatively small since it is based on fitting (i.e. VFT equation) viscosity data with many data points over a reasonably large temperature interval. The estimated error in T_g is of the order of ±2 K. This can be compared with $T_g \sim$ 400-700 K yielding a relative error of the order of ±0.003. Experimental error in determining T_L is larger. T_L is generally measured by scanning calorimetry and defined by the upper temperature limit of the observed melting endotherm. For off-eutectic alloys, this endothermic signal is spread out and may exhibit a foot-like feature on the high temperature side of the melting event. This spreading effect actually varies with the scanning rate used in the calorimetry. One may reduce the latter error by using low scan rates (~ 5 K/min. or lower) as in ref. [1]. A typical

error in T_L is estimated to be ± 5 K compared with T_L ~ 800-1200 K yielding a typical relative error of ~0.005. These two independent errors combined to give a standard error in the resulting dimensionless t_{rg} of order σ_t ~ 0.0058, or about 0.006.

The experimental error in determining Angell's parameter m arises from errors in measuring the equilibrium liquid viscosity $\eta(T)$-curve near and above T_g. Contributing to this error are (1) systematic instrumental errors, (2) errors arising from the liquid not reaching equilibrium [66], (3) or errors arising from the onset of crystallization of the liquid [68]. A complete assessment of these errors is beyond the scope of the present work. For cases where $\eta(T)$-data are reported by independent investigators, one can compare m-values obtained from independent studies. In such cases, the reported m-values are found to typically scatter by roughly ±3 around the average m value. We use this as an estimate of the standard error in m, σ_m ~ 3.

Finally, the experimental error in the reported d_{max} of a given alloy depends greatly on the effort of the reporting experimenter to establish the upper bound for the maximum rod diameter, maximum ribbon thickness, etc. for obtaining an amorphous sample. This often requires that a quenched sample be considerably overheated (often 300-600 C above T_L) or fluxed. The overheating effect is attributed to the melting of oxide inclusions [50,56]. In the case of many metal-metalloid glasses, fluxing the melt (e.g. with boron oxide) is observed to significantly reduce heterogeneous nucleants and increase GFA. In this work, the values of d_{max} in Table I were taken to be the largest reported values since those best represent intrinsic GFA of the alloy. The accuracy of the d_{max} values may vary with the details of each experimental investigation. For example, the results of silica tube water quenching with increments of 1mm in d reported in ref. [1] establish d_{max} of each alloy with an accuracy of the order of ~10% when carried out at high overheating. In other reports d_{max} values are obtained using fluxing and overheating methods [1, 26-29,32]. The lack of systematic quenching in tubes of varying d leads to uncertainty of at least ~15-20% in typical d_{max} values. Metal mold casting gives

values of d_{max} having a typical error of roughly 15%, provided that sufficient sample overheating is employed. The tendency of the quenched sample to lose contact with the mold due to differential contraction during cooling often leads to decreased values of d_{max}. Measurements of the critical ribbon thickness in variable speed melt spinning of metallic glasses likely have errors of at least ~10-20% [5]. Based on the above considerations, a typical relative error in d_{max}, for our database is taken to be ~15%, i.e. $(\sigma_{dmax}/d_{max}) \sim 0.15$.

The above error estimates in t_{rg}, m, and d_{max} were used in Eq. (3) of the main article to estimate the contribution from experimental uncertainty to the misfit between the prediction of log (d_{max}^2) based on Eq. (2) and the actual experimental data for log (d_{max}^2). The error bars in Fig.3 of the main text were determined in this manner.

GFA analysis using Senkov's parameter

Senkov [85] argued that τ_X^* should be proportional to the viscosity of an undercooled liquid at the nose temperature T^*. He combined this argument with the VFT equation to obtain a condition for glass formation:

$$F_1 = \frac{(T_g - T_o)}{0.5(T_L - T_g) - T_o} \sim \log(R_c) \qquad (6)$$

Where R_c is the critical cooling rate. The parameter F_1 can be expressed in terms of t_{rg} and the VFT parameters:

$$F_1 = \frac{2t_{rg}D}{D(1 + t_{rg}) + 16(1 - t_{rg})\ln 10} \qquad (7)$$

Senkov's parameter can be plotted for the ~40 entries in Table I to test the validity of his hypothesis. Supplementary Fig.2 shows this plot for our database. It can be compared with Figs. 1, Fig.2, and Fig. 3 of the main article. A linear regression

accounts for 88% of the variance in the GFA, significantly better than either t_{rg} or m alone (compared with Fig. 1 and Fig.2 of the main article where the respective correlation accounts for ~60% and ~50% of the variance in GFA)

GFA analysis for the ternary Pd-Ni-P system

The data used to construct Fig. 4 of the article are taken from several references [22,27,28,36,37,86-88]. For the ternary alloys $(Pd_{1-x}Ni_x)_{80}P_{20}$, Chen carried out extensive creep measurements on ribbon samples and obtained viscosity data [22,36]. He fit the data using the VFT equation and reported values for the VFT parameters D, T_0, and η_0 for x = 0.2, 0.4, 0.5, and 0.8 [22]. These parameters were used to construct

full viscosity curves to determine the rheological T_g (η = 10^{12} Pa- s) for each x. The relationship $m = [(DT_0T_g)/[\ln(10)(T-T_0)^2]_{T/Tg=1}$ is then employed to determine m for each respective x. Chen used parabolic fits to describe the composition dependence of VFT parameters. We follow this approach for the composition dependence and extrapolate m values for the binary alloy endpoints. The composition dependence obtained for m is shown in Supplementary Fig.3.

The Turnbull parameter is obtained from the rheological T_g and the liquidus temperature T_L of each alloy. Liquidus data were taken from the ASM ternary phase diagram database (vertical sections of the ternary diagram) [86] and the binary diagrams for the end points as displayed in Supplementary Fig.4. Accurate data from ref. [87] is included for the equiatomic alloy at x = 0.5. A parabolic fit is used for the x-dependence of T_L. The liquidus values vs. x are taken from this fit and combined with the rheological T_g values to determine t_{rg} vs. x. The variation of t_{rg} with x is shown in Supplementary Fig.5. The filled blue diamonds in Fig. 4 of the main article are the predicted GFA values based on Eq. (2) using the respective t_{rg} and m values. The solid blue line is a parabolic fit to the predicted values of log (d_{max}^2) for x = 0, 0.2, 0.4, 0.5, 0.8, and 1. The filled red circles in the figure are experimental values of

d_{max}^2 for the binary alloys $Pd_{80}P_{20}$ and $Ni_{80}P_{20}$ based on the fact that amorphous ribbons with thickness up to ~ 40-50 μm are reported by melt spinning. The melt spun ribbons are quenched from one side. Symmetry implies that the equivalent critical plate thickness is twice the ribbon thickness. A rod of diameter d will cool faster that a plate of thickness d by a factor of ~2 thereby giving an estimated d_{max} of about 140 μm for the critical rod diameter of the binary alloys. The open circles are taken from the GFA data of Schwarz [27,28] and that of Zeng et. al. [88]. Schwarz cast a fully glass rod of the x = 0.5 alloy [28] with a diameter of 25 mm. He noted that this is likely not the upper limit of d_{max}. He cast 10 mm diameter rods of the entire series and determined the limits of x for obtaining a glass. We take these limiting x values to indicate a d_{max} of 10 mm at the respective x. These are as shown as open circles in Fig. 4 of the main text. The final experimental GFA value is taken from Zeng et. al. [88] who reported d_{max} ~ 7mm for x = 0.75.

References for Supplementary Information

1. J.H. Na, M.D. Demetriou, M. Floyd, A. Hoff, G.R. Garrett, and W.L. Johnson, "Compositional Landscape for Glass Formation in Metal Alloys", *Proc. Nat. Acad. Sci.,* 111, 9031-9036 (2014)

2. E. Wachtel, I. Bakonyi, J. Bahle, N. Willmann, A. Lovas, A. Burgstaller, W. Socher, J. Voitlander, and H.H. Liebermann, "Magnetic Susceptibility and DSC study of the crystallization of melt quenched amorphous Ni-P alloys", *Mat. Sci. & Eng. A*, 133, 196-199 (1991)

3. Y. Nishi and A. Yoshihiro, "Viscosities of metal-metalloid alloy supercooled liquid", *Scripta Metallurgica*, 19, 1023-1028 (1985)

4. H.A. Davies, "Rapid quenching techniques and formation of metallic glasses", *Rapidly Quenched Metals III, Vol.1,* ed. by B. Cantor, (The Metals Society, London, 1978), pp. 1-21

5. Y. Waseda, S. Ueno, M. Hagiwara, and K.T. Aust, "Formation and mechanical properties of Fe- and Co-base amorphous alloy wires produce by in-rotating-water

spinning method", *Prog. in Materials Science*, 34, 149-260 (1990)
6. L.Q. Xing, D.Q. Zhao, X.C. Chen, and X.S. Chen, "Effects of melt purification on the glass forming ability and thermostability of Ni-Si-B metallic glass", Mat. Sci. and Eng., A157, 211-215 (1992)
7. T.D. Chen and R.B. Schwarz, "Bulk ferromagnetic glasses in the Fe-Ni-P-B system", Acta Mater., 49, 837-847 (2001); also T.D. Shen and R.B. Schwarz, "Bulk ferromagnetic glasses prepared by flux melting and water quenching", *Appl. Phys. Lett.,* 75, 49-51 (1999)
8. A. Dunst, D.M. Herlach, and F. Gillesen, "Formation of glassy spheres of Fe-Ni-P-B by containerless processing", Mat. Sci. and Eng., A133, 785 (1991)
9. P.M. Anderson and A.E. Lord Jr., "Viscosity of Metglas 2826 near the glass transition by rapid heating", J. Non-Cryst. Sol., 37, 219 (1980)
10. L. Zhang, X. Ma, Q. Li, J. Zhang, Y. Dong, and C. Cheng, "Preparation and properties of $Fe_{1-x}Ni_xP_{14}B_4l$ bulk metallic glasses" J. of Alloys and Compounds, 608, 79-84 (2014).
11. M.D. Demetriou, G. Kaltenboeck, J.Y. Suh, G. Garrett, M. Floyd, C. Crewdson, D. Hofmann, H. Kozachkov, A. Wiess, J. Schramm, and W.L. Johnson, "Glass steel optimized for glass-forming ability and toughness", *Appl. Phys. Lett.* 95*, 041907 (2009)*
12. V. Ponnambalam, S.J. Poon, and G.J. Shiflet, "Fe-based bulk metallic glasses with diameter larger than one centimeter", *J. Mater. Res.,* 19, 1320-1323 (2004)
13. V. Ponnambalam, S.J. Poon, G.J. Shiflet, V.M. Keppens, R. Taylor, and G. Petculescu, "Synthesis of iron-based bulk metallic glasses as non-ferromagnetic amorphous steels", *Appl. Phys. Lett.,* 83, 1131-1133 (2003)
14. X.J. Gu, S.J. Poon, G.J. Shiflet, and M. Widom, "Ductility improvement of amorphous steels: Roles of shear modulus and electronic structure", *Acta Mater.* 56, 88-94 (2011)
15. J.H. Na, M.D. Demetriou, and W.L. Johnson, "Fragility of Fe-based glasses", *Appl. Phys. Lett.,* 99, 161902 (2011)
16. H.S. Chen and D. Turnbull, "Evidence of a glass transition in a Gold-Germanium-Silicon alloy", *J. Chem. Phys.,* 48, 2560-2571 (1968)

17. H. Davies, "The kinetics of formation of a Au-Si-Ge metallic glass", *J. Non-Cryst. Sol.,* 17, 266-272 (1975)
18. N. Chen, H. Zhang, and K.F. Yao, "Formation and properties of binary Pd-Si bulk metallic glasses", *Adv. in Mat. Sci. and Eng.*, 2014, 647197 (2014)
19. We have conducted water quenching experiments using silica tubes of 1mm wall thickness in which $Pd_{84}Si_{16}$ samples were processed at 1350 C in boron oxide flux for time periods up to ~10 hours then quenched and stirred in a water bath. Glassy rods up to 2 -3 mm diameters are produced by this method.
20. Y. Nishi, N. Kayama, S. Kiuchi, K. Suzuki, and T. Masumoto, "Viscosities and glass formation of $Pd_{84}Si_{16}$ and $Pd_{78}Cu_6Si_{16}$ Alloys", J. Japan Inst. Metals, 44, 1336-1341 (1980)
21. H.S. Chen and M. Goldstein "Anomalous viscoelastic behavior of metallic glasses of Pd-Si alloys", *J. Appl. Phys.,* 43, 1642-1648 (1972)
22. H.S. Chen, "Alloying effect on the viscous flow behavior of metallic glasses", *J. Non-Cryst. Sol*., 29, 223-229 (1978)
23. J. Steinberg, A.E. Lord, L.L. Lacy, and J. Johnson, "Production of bulk amorphous $Pd_{77.5}Si_{16.5}Cu_6$ in a container-less low gravity environment", *Appl. Phys. Lett., ,* 38, 135-137 (1981)
24. H.S. Chen and D. Turnbull, "Formation, stability, and structure of Pd-Si based alloy glasses", *Acta Metallurgica*, 17, 1021-1031 (1969)
25. D.H. Yu, L.I. Yang and K.F. Yao, "Preparation of a Pd-Cu-Si metallic glass with a diameter up to 11mm", *Chin. Phys. Lett.,* 27m 126101 (2010)
26. D. Granata, E. Fischer, V. Wessels, and J.R. Loeffler, "Fluxing of Pd-Si-Cu bulk metallic glass of and the role of cooling rate and purification", *Acta Mater.,* 71, 145-152 (2014)
27. Y. He, R.B. Schwarz, and J.I. Archuleta, "Bulk Glass Formation in the Pd-Ni-P System", Appl. Phys. Lett., 69, 1861-1863 (1996)
28 Y. He, T. Shen, and R.B. Schwarz, "Bulk Amorphous Metallic alloys; Synthesis by Fluxing and Properties", Metallurgical and Materials Transactions A, 29A, 1795-1804 (1998)

29. H.S. Chen, J.T. Krause, and E. Coleman, "Elastic Constants, Hardness, and their Implications for Flow Properties of Metallic Glasses", J. Non-Crystalline. Solids, 18, 157-171 (1975)
30. H.S. Chen, "The Glass Transition Temperature in Metallic Glasses: Effects of Atomic Sizes and the Heats of Mixing", Acta Metallurgica, 22, 897-900 (1974)
31. W.L. Johnson, M.D. Demetriou, J.S. Harmon, M.L. Lind, and K. Samwer, "Rheology and ultrasonic properties of metallic glass-forming liquids: A potential energy landscape perspective", *MRS Bulletin*, 32, 644-650 (2007)
32. A.J. Drehman, A.L. Greer, and D. Turnbull, "Bulk formation of a metallic glass $Pd_{40}Ni_{40}P_{20}$, *Appl. Phys. Lett.*, 41, 716-717 (1982)
33. H.W. Kui, A.L. Greer, and D. Turnbull, "Formation of bulk metallic glass by fluxing", *Appl. Phys. Lett.*, 45, 615-616 (1984)
34. I. Gallino, J. Schroers, and R. Busch, "Kinetic and thermodynamic studies of the fragility of bulk metallic glass forming liquids", *J. Appl. Phys.*, 108, 063501 (2010)
35. I.R. Lu, G. Wilde, G.P. Gorler, and R. Willnecker, "Thermodynamic studies of Pd-based glass forming alloys", *J. Non-Cryst. Sol.,* 250, 577-581 (1999).
36. H.S. Chen, "Method of evaluating viscosities of metallic glasses from rates of thermal transformations", *J. Non-Cryst. Sol.,* 27, 257-260 (1978)
37. C.A. Volkert and F. Spaepen, "Viscosity and Structural Relaxation in $Pd_{40}Ni_{40}P_{19}Si_{1}$", *Mat. Sci. & Eng.,* 97, 449-452 (1988)
38. N. Nishiyama and A. Inoue, "Flux treated Pd-Cu-Ni-P amorphous alloy having low critical cooling rate", *Mat. Trans. JIM,* 38, 464-472 (1997)
39. J.C. Qiao, S. Cardinal, J.M. Pelletier, "Insight on the process-ability of metallic glasses by thermomechanical analysis and dynamic mechanical analysis", J. Alloys and Compounds, 628, 357 (2015)
40. N. Nishiyama, K. Takenaka, H. Miura, N. Saido, Y. Zeng, and A. Inoue, "Worlds largest glassy alloy ever made", *Intermetallics*, 30, 19-24 (2012)
41. N. Nishiyama, K. Takenaka, H. Miura, N. Saido, Y. Zeng, and A. Inoue,"Stabiity and nucleation behavior of glass forming Pd-Cu-Ni-P alloy with critical cooling rate of 0.067 K/s", *Intermetallics*, 10, 1141-1147 (2002)

42. H.Kato, T. Wada, M. Hasegawa J. Saida, A. Inoue, and H.S. Chen, "Fragility and thermal stability of Pt-based and Pd-based bulk glass forming liquids and their correlation with deformability, Scripta Materialia, 54, 2023-2027 (2006)
43. J.F. Loeffler, J. Schroers, and W.L. Johnson, "Time temperature transformation diagram and microstructures of bulk glass forming $Pd_{40}Cu_{30}Ni_{10}P_{20}$", *Appl. Phys. Lett., 77, 681-683 (2000)*
44. K. Russew, L. Stojanova, S. Yankova, E. Fazakas, and L.K. Vargo, "Thermal behavior and melt fragility number of $Cu_{100-x}Zr_x$ glassy alloys in terms of crystallization and viscous flow", *J. of Physics*, Conf. Series 144, 012094 (2009)
45. Q. Wang, L. –M. Wang, M.Z. Ma, S. Binder, T. Volkmann, D.M. Herlach, J.S. Wang, Q.G. Xue, Y.J. Tian, and R.P. Liu, "Diffusion-controlled crystal growth rate in deeply undercooled $Zr_{50}Cu_{50}$ melt on approaching the glass transition", *Phys. Rev. B*, 83, 014202 (2011)
46. W.H. Wang, J.J. Lewandowski, and A.L. Greer, "Understanding the glass forming ability of $Cu_{50}Zr_{50}$ alloys in terms of a metastable eutectic", *J. Mater. Res.,* 20, 2307-2313 (2005)
47. X.H. Lin and W.L. Johnson, "Formation of Ti-Zr-Cu-Ni bulk metallic glasses", *J. Appl. Phys.,* 78, 6514-6519k (1995); also see Ph.D. thesis, X.H. Lin, "Glass forming ability…", California Institute of Technology, (1997)
48. H. Choi-Yim, R. Busch, and W.L. Johnson, "The effect of Si on the glass forming ability of $Cu_{47}Ti_{34}Zr_{11}Ni_8$ alloy", *J. Appl. Phys.,* 83, 7993-7997 (1998)
49. S.C. Glade and W.L. Johnson, "Viscous flow of the $Cu_{47}Ti_{34}Zr_{11}Ni_8$ glass forming alloy", *J. Appl. Phys.,* 87, 7249-7254 (2000)
50. X.H, Lin, W.K. Rhim, and W.L. Johnson, "Effect of oxygen impurity on the crystallization of an undercooled bulk glass forming alloy", Mater. Trans. JIM, 38, 473-477 (1997)
51. C.C. Hays, J. Schroers, U. Geyer, S. Bossuyt, N. Stein, and W.L. Johnson, "Glass forming ability of Zr-Nb-Ni-Cu-Al bulk metallic glasses", Mater. Sci. Forum, 343, 103-108 (2000)

52. S.C. Glade, D.S. Lee, R. Wunderlich, and W.L. Johnson, "AC modulation calorimetry of undercooled liquid $Cu_{47}T_{34}Zr_{11}Ni_8$, $Zr_{57}Nb_5Ni_{12.6}Al_{10}Cu_{15.4}$ – An MSL-1 experiment using TEMPUS", MRS Symp. Proc., 551, 219-225 (1999)
53. S.C. Glade, R. Busch, D.S. Lee, W.L. Johnson, R.K. Wunderlich, and H.J. Fecht, "Thermodynamics of $Cu_{47}Ti_{34}Zr_{11}Ni_8$, $Zr_{52.5}Cu_{17.9}Ni_{14.6}Al_{10}Ti_5$, and $Zr_{57.5}Cu_{15.4}Ni_{12.6}Al_{10}Nb_5$ bulk metallic glas alloys, *J. Appl. Phys.,* 87, 7242-7248 (2000)
54. Z. Evenson, S. Raedersdorf, I. Gallino, and R. Busch, "Equilibrium viscosity of Zr-Cu-Ni-Al-Nb bulk metallic glasses", *Scripta Mat.*, 63, 573-576 (2010); also Z. Evenson, T. Schmitt, M. Nicola, I. Gallino, and R. Busch, "High temperature melt viscosity and fragile to strong transition in $C_{47}Ti_{34}Zr_{11}Ni_8$ and Zr-Cu-Ni-Al-Nb(Ti) bulk metallic glasses", in 4th Int. Symposium on Slow Dynamics in Complex Systems, AIP Conf. Proc. 1518, 197-205 (2013); also Z. Evenson, "On the thermodynamic and kinetic properties of bulk glass forming metallic systems", Ph.D. Doctoral thesis, University of Saarbrucken, Germany (submitted June 2012)
55. C.C. Hayes, J. Schroers, W.L. Johnson, T.J. Rathz, R.W. Hyers, J.R. Rogers, and M.B. Robinson, "Vitrification and determination of the crystallization time scale of the bulk metallic glass forming liquid $Zr_{58.5}Nb_{2.8}Cu_{15.7}Ni_{12.8}Al_{10.3}$", *Appl. Phys. Lett.,* 79, 1605-1607 (2001).
56. S. Mukherjee, "Study of crystallization behavior, kinetics, and thermodynamics of bulk metallic glasses using non-contact electrostatic levitation technique", Ph.D. thesis, Dept. of Materials Science, California Institute of Technology (submitted 2006)
57. S. Mukherjee J. Schroers, W.L. Johnson, and W.K. Rhim, "Influence of kinetic and thermodynamic factors on the glass forming ability of Zr-based bulk amorphous alloys, *Phys. Rev. Lett.,* 94, 245501 (2005)
58. J.C. Qiao, R. Casalini, and J.M. Pelletier, "Main alpha relaxation and excess wing in $Zr_{50}Cu_{40}Al_{10}$ bulk metallic glass investigated by mechanical spectroscopy", J. Non-Cryst. Sol., 407, 106 (2015)
59. Y. Yokoyama, K. Fakaura, and A. Inoue, "Cast structure and and mechanical

properties of Zr-Cu-Ni-Al bulk glassy alloys", Intermetallics, 10, 1113 (2002)
60. A. Inoue and A. Takeuchi, "Recent development and application products of bulk glassy alloys", *Acta Materialia*, 59, 2243-2267 (2011)
61. A. Peker and W.L. Johnson, "A highly processable metallic glass $Zr_{41.2}Ti_{13.8}Cu_{12.5}Ni_{10}Be_{22.5}$", *Appl. Phys. Lett.,* 63, 2342-2344 (1993)
62. *unpublished results*, A. Peker and W.L. Johnson, casting of 1", 2", and 3" rods of Vitreloy 1 at Retech. Corp. (CA) by plasma melting and pouring into water cooled copper molds. 1 ft. long Rods of 1" and 2" diameter fully amorphous.
63. Y.J. Kim, R. Busch, W.L. Johnson, A.J. Rulison, and W.K. Rhim, "Experimental determination of the time-temperature-transformation diagram of the undercooled liquid $Zr_{41.2}Ti_{13.8}Cu_{12.5}Ni_{10}Be_{22.5}$ alloy using containerless electrostatic levitation processing", *Appl. Phys. Lett.,* 68, 1057-1059 (1996).
64. R. Busch, E. Bakke, and W.L. Johnson, "Viscosity of the supercooled liquid and relaxation at the glass transition of the $Zr_{46.75}Ti_{8.25}Cu_{7.5}Ni_{10}Be_{27.5}$ bulk metallic glass forming alloy", *Acta Mater.,* 46, 4725-4732 (1998)
65. R. Busch, E.J Bakke, and W.L Johnson, "On the glass forming ability of bulk metallic glasses", *Mater. Sci. Forum*, 235, 327-335 (1997)
66. R. Busch and W.L. Johnson, 'The kinetic glass transition of the $Zr_{46.75}Ti_{8.25}Cu_{7.5}Ni_{10}Be_{27.5}$ bulk metallic glass former-supercooled liquids on a long time scale", Appl. Phys. Lett., 72, 2695 (1998)
67. T.A. Waniuk, "Viscosity and Crystallization in a series of Zr-based bulk amorphous alloys", Ph.D. thesis, California Institute of Technology, (submitted April, 2004)
68. T.A. Waniuk, R. Busch, A. Masuhr, and W.L.Johnson, "Equilibrium viscosity of the $Zr_{41.2}Ti_{13.8}Cu_{12.5}Ni_{10}Be_{22.5}$ bulk metallic glass forming liquid and viscous flow during relaxation, phase separation, and crystallization", *Acta Mater.*, 46, 5229-5236 (1998)
69. R. Busch, W. Liu, and W.L. Johnson, "Thermodynamics and Kinetics of the $Mg_{65}Cu_{25}Y_{10}$ bulk metallic glass forming liquid", *J. Appl. Phys.,* 83, 4134-4141 (1998)
70. Q. Zheng, J. Xu, and E. Ma, "High glass-forming ability correlated with fragility of Mg-Cu(Ag)-Gd alloys", *J. Appl. Phys.,* 102, 113519 (2007)

71. Y.C. Chang, J.C. Huang, C.W. Tang, C.I. Chang, and J.S.C. Jang, "Viscous flow behavior and workability of Mg-Cu(Ag)-Gd bulk metallic glasses", *Mat. Trans.,* 49, 2605-2610 (2008)
72. H. Ma and H.J. Fecht, "Thermodynamic and kinetic fragilities of Mg-based bulk metallic glass forming liquids", J. of Mater. Res., 23, 2816-2820 (2008)
73. Y. Kawamura, T. Nakamura, H. Kato, H. Mano, and A. Inoue, "Newtonian and Non-Newtonian viscosity of supercooled liquid metallic glass", *Mat. Sci. & Eng. A*, 304-306, 674-678 (2001)
74. W. Zhang, F. Jia, and A. Inoue, "Formation and properties of new La-based bulk glassy alloys with diameter up to 12 mm" , *Mater. Trans., JIM*, 48, 68-73 (2007)
75. Y. Ji, S. Pang, C. Ma, and T. Zhang, "Formation of La-Al-Ni-Cu-Fe bulk metallic glasses with high glass forming ability", Int. J of Modern Phys. B, 24, 2314-2319 (2010)
76. Q.K. Jiang, G.Q. Zhang, L Yang, X.D. Wang, K. Saksl, H. Franz, R. Wunderlich, H.J. Fecht, and J.Z. Jiang, "La-based bulk metallic glasses with critical diameter up to 30 mm", Acta Mater., 55, 4409-4417 (2007)
77. G.J. Diennes and H.F. Klemm, "Theory and Application of the Parallel Plate Plastometer", J. Appl. Phys., 17, 458 (1946)
78. C.A. Angell, "Formation of glasses from liquids and biopolymers", Science, 267, 1924-1935 (1995)
79. M. D. Demetriou *et al.*, "Cooperative shear model for the rheology of glass forming metallic liquids". *Phys Rev Lett* **97**, 065502 (2006).
80. S. Mukherjee, W.L. Johnson, and W.K. Rhim, "High temperature measurement of surface tension and viscosity of bulk metallic glass forming alloys using the drop oscillation technique", 86, 014104 (2005)
81. H. Shibatu, S. Nishihata, H.Ohta, S. Suzuki, Y. Waseda, M. Imafukua, J. Saida, and A. Inoue, "Thermal diffusivity of Zr-based bulk metallic glass alloys in the liquid state", Mat. Trans. JIM, 48, 886-888 (2007)

82. M. Yamasaki, S. Kagao, and Y. Kawamura, "Thermal diffusivity and conductivity of supercooled Zr-Ti-Cu-Ni-Be metallic glass", Appl. Phys. Lett., 84, 4653-4655 (2004)

83. J. Schroers, S. Bossuyt, W.K. Rhim, J.Z. Li, Z.H. Zhou, and W.L. Johnson, "Enhanced temperature uniformity by tetrahedra laser heating", Rev. of Sci. Instr., 75, 4523-4527, (2004)

84. S. Pogatscher, P.J. Uggowitzer, and J.F. Loeffler, "In-situ probing of metallic glass formation and crystallization upon heating and cooling via fast differential scanning calorimetry", Appl. Phys. Lett., 104, 251908 (2014)

85. O.N. Senkov, "Correlation between fragility and glass forming ability of metallic alloys", Phys. Rev. B,, 76, 104202 (2007)

86. E. Wachtel, H. Haggag, T. Godecke, and B. Predel, the Ni-P-Pd phase diagram, ASM Alloy Phase Diagrams Database, P. Villars, editor-in-chief, H. Okamoto and K. Cenzual, section editors; http://www1.asminternational.org/AsmEnterprise/APD, ASM International, Materials Park, OH, (2006)

87. R. Willnecker, K. Wittmann, and G.P. Gorler, "Undercooling measurements and heat capacity investigations of Pd-Ni-P melts", J. Non-Cryst. Sol., 156-158, 450-454 (1994)

88. Y.Q. Zeng, A. Inoue, N. Nishiyama, and M.W. Chen, "Ni-rich Ni-Pd-P metallic glasses with significantly improved glass forming ability", Intermetallics, 18, 1790-1793 (2010)
